# Autophagy inhibition elicits emergence from metastatic dormancy by inducing and stabilizing Pfkfb3 expression

**DOI:** 10.1038/s41467-019-11640-9

**Published:** 2019-08-14

**Authors:** Alyssa La Belle Flynn, Benjamin C. Calhoun, Arishya Sharma, Jenny C. Chang, Alexandru Almasan, William P. Schiemann

**Affiliations:** 1 Case Western Reserve University, Department of Pharmacology, Cleveland, OH 44106 USA; 2 Department of Pathology and Laboratory Medicine, University of North Carolina, Chapel Hill, NC 27599 USA; 3 Department of Cancer Biology, Lerner Research Institute, Cleveland Clinic, Cleveland, OH 44106 USA; 4 Houston Methodist Research Center, Houston, TX 77030 USA; 5 Case Comprehensive Cancer Center, Case Western Reserve University, Cleveland, OH 44106 USA

**Keywords:** Breast cancer, Metastasis

## Abstract

Breast cancer stem cells (BCSCs) are unique in their ability to undergo unlimited self-renewal, an essential process in breast cancer recurrence following metastatic dormancy. Emergent metastatic lesions were subjected to microarray analysis, which identified 6-phosphofructo-2-kinase/fructose-2,6-biphosphatase 3 (Pfkfb3) as a differentially expressed gene coupled to metastatic recurrence. Here, we report that elevated Pfkfb3 expression correlates with the appearance of aggressive breast cancers and reduces relapse-free survival, as well as enhances BCSC self-renewal and metastatic outgrowth. We observe an inverse relationship between Pfkfb3 expression and autophagy, which reduces Pfkfb3 expression and elicits cellular dormancy. Targeted depletion of Atg3, Atg7, or p62/sequestosome-1 to inactivate autophagy restores aberrant Pfkfb3 expression in dormant BCSCs, leading to their reactivation of proliferative programs and outgrowth. Moreover, Pfkfb3 interacts physically with autophagy machinery, specifically the UBA domain of p62/sequestosome-1. Importantly, disrupting autophagy and this event enables Pfkfb3 to drive dormant BCSCs and metastatic lesions to recur.

## Abstract (web summary)

Cancer cells can use autophagy as a mechanism of surviving from external stresses. Here, the authors show that Pfkfb3, a glycolytic gene, is expressed in metastatic breast cancer cells but not in dormant cells that demonstrate features of autophagy.

## Introduction

Among women, breast cancer is the second leading cause of cancer-associated deaths, claiming the lives of more than 40,000 women in the United States in 2015 (ref. ^1^). Nearly 90% of these breast cancer-associated deaths were due to metastasis, a clinically incurable disease that is poorly understood^2^. Evidence suggests that even in the earliest stages of breast cancer, disseminated tumor cells (DTCs) are shed from the growing tumor, enter the bloodstream, extravasate, and colonize distant metastatic sites^3,4^. While many cells escape the primary tumor, only those capable of tumor initiation (i.e., breast cancer stem cells (BCSCs)) ultimately give rise to the formation of overt metastasis^5^. Upon colonization of distant metastatic sites, BCSCs can enter a state of metastatic dormancy, thereby halting their proliferation and activating cellular stress response pathways while remaining metabolically active^6–9^. Recent evidence suggests that dormant phenotypes are malleable and manipulation of intrinsic and/or extrinsic factors can reverse dormant phenotypes and reinitiate proliferative programs in vivo^10,11^. While ~62% of all deaths attributed to breast cancer occur 5 years or more following clinical disease remission, the mechanisms that render dormant BCSCs competent to reactivate proliferative programs remain unknown^12^.

During the colonization of metastatic sites, BCSCs can activate macroautophagy (henceforth referred to as autophagy), a process by which cells recycle macromolecules as a means to overcome environmental stressors (e.g., nutrient deprivation) and ensure for their survival^7^. The polyubiquitin-binding protein, p62/sequestosome-1 (SQSTM1) is a vital component of the autophagy machinery that is degraded during autophagy^13^. Tumor cells with deficient autophagic processes can accumulate p62/SQSTM1, an event that alters its function and contributes to tumorigenesis^13^. Recent findings suggest that autophagy functions in maintaining cancer stem cell (CSC) phenotypes, particularly their acquisition of chemoresistant phenotypes^7,13,14^.

Along these lines, the critical glycolysis mediator 6-phosphofructo-2-kinase/fructose 2,6-biphosphatase 3 (Pfkfb3) has also been linked to autophagic processes^15–17^. Pfkfb3 is one of four Pfkfb isoforms that regulates the rate of glycolysis by generating fructose 2,6-bisphosphate (Fru-2,6-P2)^18^. Besides its glycolytic and autophagic activities, Pfkfb3 also plays important roles in regulating several cell behaviors, including (i) stimulating endothelial cell vessel sprouting in response to hypoxia^19^; (ii) promoting cell cycle progression through the G1 and S phases^20^; and (iii) suppressing apoptosis^21^. The Warburg Effect refers to the ability of cancer cells to preferentially utilize glycolysis as an energy source even under aerobic conditions, thereby contributing to cancer initiation and progression^22^. Accordingly, upregulated Pfkfb3 expression is evident in numerous human cancers as compared to corresponding normal tissue^23^. Given the known roles of Pfkfb3, we hypothesized that Pfkfb3 could play a critical role in promoting dormant metastatic cells to reactivate proliferative programs and recur.

Here, we show that Pfkfb3 expression is upregulated in metastatically competent cells and predicts for reduced relapse-free survival (RFS). Importantly, heterologous expression of Pfkfb3 in dormant BCSCs significantly increased their frequency and capacity for self-renewal, and conversely, depleting Pfkfb3 expression in metastatic BCSCs significantly reduced their survival and outgrowth proficiency both in vitro and in vivo. Pfkfb3 expression and autophagy exhibited an inverse relationship, such that dormant breast cancer cells displayed a Pfkfb3^Low^Autophagy^High^ phenotype that gave way to a Pfkfb3^High^Autophagy^Low^ phenotype in metastatic breast cancer cells. As such, pharmacologic and genetic inactivation of autophagy dramatically upregulated Pfkfb3 expression in dormant breast cancer cells, thus enabling their outgrowth both in vitro and in vivo. Collectively, our findings indicate that emergence from metastatic dormancy and subsequent disease recurrence reflects a phenotypic shift from a Pfkfb3^Low^Autophagy^High^ to a Pfkfb3^High^Autophagy^Low^ profile, suggesting that monitoring tumor Pfkfb3 and autophagy status may offer insights into patient long-term survival and response to therapy.

## Results

### Characterization of dormant BCSC growth and self-renewal

To identify drivers capable of overcoming metastatic dormancy, we utilized the D2-hyperplastic alveolar nodule (HAN) progression series, a well-established model of breast cancer dormancy that comprises dormant D2.OR cells and metastatic D2.A1 cells originally derived from BALB/c mice^24–26^. Accordingly, D2.OR cells express numerous senescence-associated genes not expressed by their metastatic D2.A1 counterparts (Supplementary Fig. 1a). Although both cell lines exhibit identical growth rates in traditional two-dimensional (2D)-culture systems^10,24–27^, D2.OR cells readily acquire dormant phenotypes when: (i) cultured in three-dimensional (3D) cultures comprising reconstituted basement membrane extracts that mimic the extracellular matrix composition of the pulmonary microenvironment (Supplementary Fig. 1b), and (ii) inoculated into the lungs via the lateral tail veins of syngeneic BALB/c mice^10,28,29^. While D2.OR and D2.A1 cells both extravasate into lung tissue, only D2.A1 cells readily colonize the lung and form large metastatic lesions that rapidly kill mice^24,25^. In contrast, D2.OR cells readily enter a state of dormancy in the lungs of BALB/c mice^30^.

**Fig. 1:**
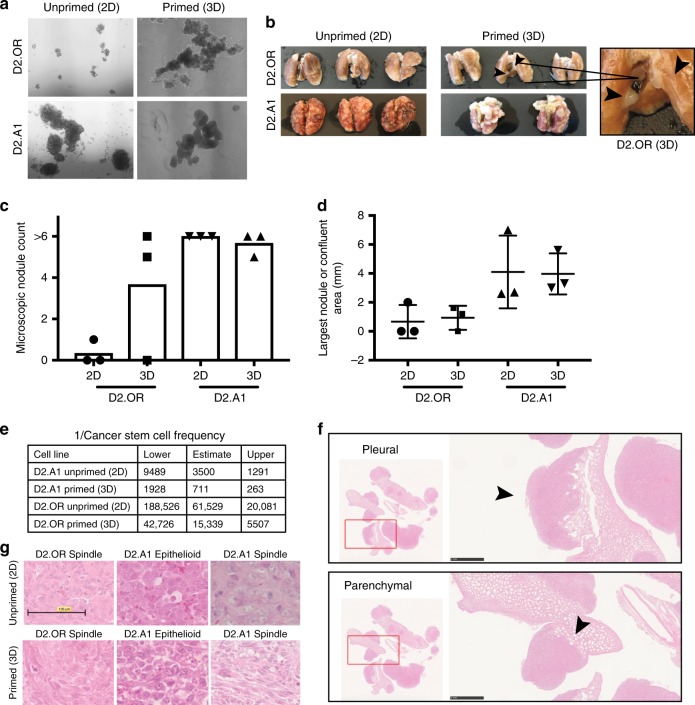
Characterization of dormant BCSC growth and self-renewal. **a** Mammosphere assay of primed and unprimed D2.OR and D2.A1 cells (1 × 10^4^ cells/well in a six-well plate). **b** Excised lung tissue from mice inoculated with D2.OR (2D), D2.OR (3D), D2.A1 (2D), or D2.A1 (3D) cells (1 × 10^6^ cells/mouse). D2.OR (3D) lungs exhibit small tumor nodules as shown by the black arrows (and inset). D2.A1 (2D) and D2.A1 (3D) have extensive tumor nodules covering the outer pleural surface (*n* = 3 mice/group). **c** Microscopic nodule count from lung tumors in **b**. **d** Largest nodule or confluent area (as measured in millimeters (mm) by NanoZoomer Digital Pathology viewer software). **e** Estimated stem cell frequencies of D2.A1 and D2.OR 2D and 3D cells when inoculated into the mammary fat pads of BALB/c mice (100, 1000, or 10,000 cells/mouse injected for D2.A1 cells and 500, 5000, or 50,000 cells/mouse injected for D2.OR cells; *n* = 5 mice/group). Estimated stem cell frequencies at 95% confidence interval were generated using http://bioinf.wehi.edu.au/software/elda/. **f** Hematoxylin and eosin-stained D2.A1 pleural and parenchymal morphologies. Scale bar = 1 mm. **g** Hematoxylin and eosin-stained dormant D2.OR and metastatic D2.A1 lung tumor nodules with differential morphologies derived from BALB/c mice. Scale bar = 100 μM

The sequential propagation of these same D2.HAN derivatives from 2D-plastic to a 4 to 5-day span in 3D cultures (i.e., priming) dramatically drove the expansion and aggressiveness of BCSCs both in vitro (i.e., mammospheres; Fig. 1a) and in vivo (i.e., lung metastases; Fig. 1b). Indeed, in vitro breast cancer cell priming significantly expanded the growth and self-renewal of dormant D2.OR BCSCs as evidenced by serial mammosphere assays where primed cells produced larger and more numerous spheres relative to their unprimed counterparts (Fig. 1a). When inoculated into the lateral tail veins of BALB/c mice, primed breast cancer cells formed macroscopic pulmonary tumor nodules that were not evident in the lungs of mice inoculated with unprimed D2.OR cells (Fig. 1b). We also show that primed D2.OR-derived tumors form more numerous microscopic nodules (Fig. 1c) and that metastatic D2.A1 cells form tumors that are larger than their dormant D2.OR counterparts (Fig. 1d). Furthermore, in vivo extreme limiting dilution assays (ELDA) of primed and unprimed dormant and metastatic cells clearly detected increased stem cell frequencies (Fig. 1e) and decreased tumor latencies (Supplementary Fig. 2a, b) in primed populations as compared to their unprimed counterparts. Finally, it should be noted that metastatic foci derived from D2.A1 cells exhibited two distinct tumor morphologies: (i) tumor nodules of epithelioid morphology were exclusively located within the pulmonary parenchyma, and (ii) tumor nodules comprised spindle-shaped cells were restricted to the pulmonary pleural surface (Fig. 1f, g). As expected, unprimed D2.OR cells acquired dormant phenotypes in the lungs of BALB/c mice, resulting in the formation of small micro-nodules. In stark contrast, primed D2.OR cells were capable to varying extents of forming macroscopic and microscopic spindle-shaped tumor nodules within both the parenchymal and pleural lung compartments. Collectively, these findings suggest that a switch in metastatic dormancy may reflect differences in the location of the metastatic niche within the lung, as well as alterations in the resultant morphology exhibited by colonizing BCSCs.

### Pfkfb3 expression correlates with metastatic dormancy escape

**Fig. 2:**
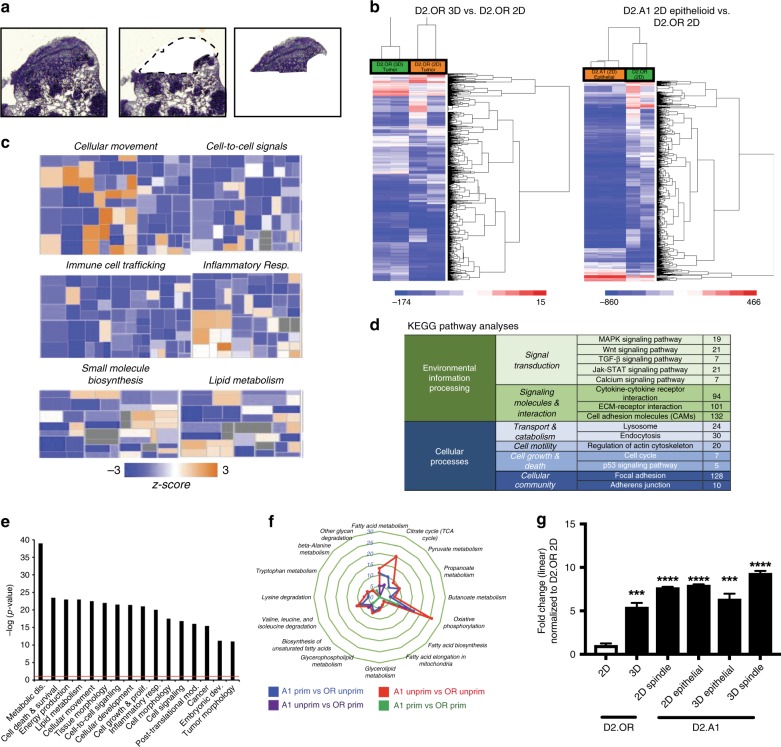
Aberrant Pfkfb3 expression correlates with emergence from metastatic dormancy. **a** Laser capture microdissection of cresyl violet-stained 12-μm-thick murine lung tumors from Fig. 1b at ×1.25 magnification. **b** Heatmap derived from microarray analysis with threefold differences between each evaluated group (*n* = 3 mice/group). **c** Ingenuity Pathway Analysis (IPA) hierarchical heatmap derived from Downstream Effects Analysis. *Z*-score represents activation of each biological function where blue represents decreased function and orange represents increased function. Fold change cutoff of 3. **d** IPA-derived pathway analysis identified pathways significantly altered (threefold change) between dormant D2.OR and metastatic D2.A1 derived pathways. **e** KEGG Pathway analysis reveals major pathways altered between dormant D2.OR and metastatic D2.A1 cells (threefold change cutoff). **f** Spider plot depicts the number of genes altered more than threefold between comparison groups in genes belonging to the identified metabolic pathways. **g** Pfkfb3 expression in samples analyzed via Affymetrix GeneChip Mouse 2.0 ST microarrays

To evaluate the molecular mechanisms that underlie the differences in the morphology and outgrowth proficiency of these metastatic D2-HAN nodules, we performed laser capture microdissection to procure essentially pure tumor samples comprising the aforementioned metastatic morphologies (Fig. 2a). In doing so, total RNA was isolated and differences in gene expression were identified using Affymetrix GeneChip Mouse 2.0 ST microarrays. The resulting probe intensities were normalized via Robust Multi-array Average, at which point fold-change values were analyzed via Ingenuity Pathway Analysis and KEGG analysis to identify potential pathways capable of mediating escape from metastatic dormancy. Comparing unprimed D2.OR cells to their primed counterparts revealed 295 genes (e.g., 244 downregulated and 51 upregulated) that displayed a ≥2-fold difference in gene expression and an A-NOVA *p* value of <0.05 between groups (Fig. 2b). Among the most significantly altered canonical pathways evident in dormant versus metastatic tumor nodules were those genes involved in cell movement, cell–cell signaling, immune cell trafficking/inflammation, and cell metabolism (Fig. 2c–f). Given the large number of differentially expressed genes identified by microarray analysis, we identified potential mediators of metastatic recurrence (i.e., genes that promote escape from dormancy) by distilling our analyses to genes whose expression increased in a manner associated with increased BCSC frequency (Fig. 1e; unprimed D2.OR<primed D2.OR<unprimed D2.A1<primed D2.A1). Included among the most differentially expressed mRNAs evaluated in this microarray analysis was the critical metabolic regulator, 6-phosphofructo-2-kinase/fructose 2,6-biphosphatase 3 (Pfkfb3). Indeed, Pfkfb3 expression was significantly elevated (by 4–8-fold) in metastatic tumors relative to their dormant counterparts (Fig. 2g). Collectively, these findings suggest a role for Pfkfb3 regulating metastatic relapse and recurrence by disseminated breast cancer cells.

### Pfkfb3 is correlated with reduced RFS

**Fig. 3:**
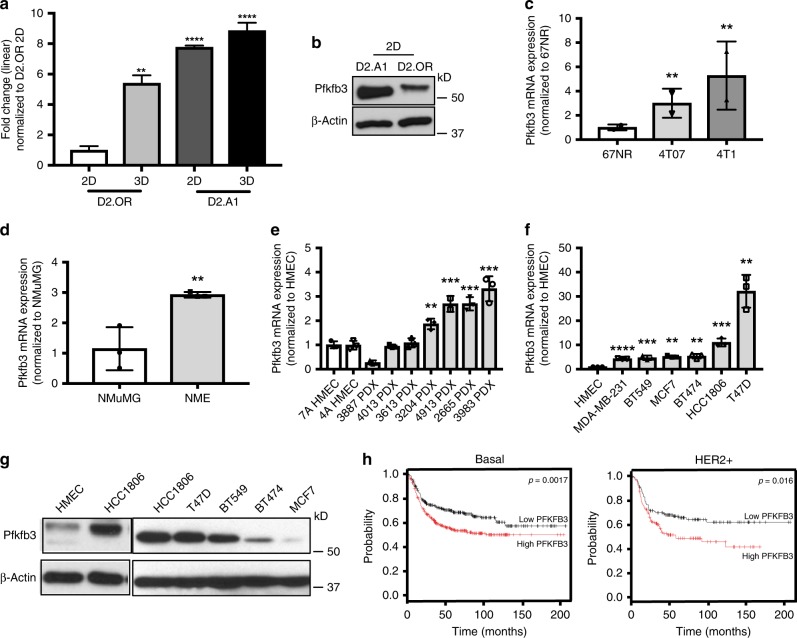
Aberrant Pfkfb3 expression correlated with disease progression and reduced relapse-free survival in human breast cancers. **a** Expression of Pfkfb3 mRNA in unprimed and primed D2.OR and D2.A1 cells as indicated. Data are the mean (±SD; *n* = 3) Pfkfb3 transcript levels normalized to D2.OR 2D. **P ≤ 0.01 and ****P ≤ 0.0001. This experiment was independently repeated for a total of three experiments, all with similar results. **b** Analysis of Pfkfb3 protein in unprimed D2.A1 and D2.OR cells. **c** mRNA expression across the 4T1 progression series. Data are the mean (±SD; *n* = 3) Pfkfb3 transcript levels normalized to 67NR cells. **P ≤ 0.01). This experiment was independently repeated for a total of three experiments, all with similar results. **d** Transformed NME cells harbor higher Pfkfb3 expression than NMuMG cells. Data are the mean (±SD; *n* = 3) Pfkfb3 transcript levels normalized to NMuMG cells. **P ≤ 0.01. This experiment was independently repeated for a total of three experiments, all with similar results. **e** High expression of Pfkfb3 is common in PDX models compared to normal HMECs. Data are the mean (±SD; *n* = 3) Pfkfb3 transcript levels normalized to HMECs. **P ≤ 0.01 and ***P ≤ 0.001. This experiment was independently repeated for a total of three experiments, all with similar results. **f** PFKFB3 mRNA expression by RT-PCR in human tumor cell lines. Data are the mean (±SD; *n* = 3) Pfkfb3 transcript levels normalized to HMECs. **P ≤ 0.01, ***P ≤ 0.001, and ****P ≤ 0.0001. This experiment was independently repeated for a total of three experiments, all with similar results. **g** PFKFB3 protein expression by immunoblot with β-actin used as a loading control. **h** Relapse-free survival analysis of 618 basal and HER2+ patients stratified by PFKFB3 expression levels. The expression range of the probe was between 225–5404 and the cutoff value for high PFKFB3 expression was 921 (https://www.kmplot/analysis.com)

The known biological functions of Pfkfb3 include critical roles in promoting glycolysis^20,21^, and in stimulating vessel sprouting^19^, cell survival^21^, and cell proliferation^21^, events that are essential for ensuring the survival and recurrence of metastatic breast cancers. These findings, together with those above, raised the possibility that aberrant Pfkfb3 expression correlates with breast cancer tumor progression and the acquisition of aggressive phenotypes. To test this supposition, we monitored the expression patterns of Pfkfb3 in normal and malignant murine and human breast samples. In doing so, we observed Pfkfb3 mRNA (Fig. 3a) and protein expression (Fig. 3b) to be significantly upregulated in metastatic D2.A1 cells as compared to their dormant D2.OR counterparts. Moreover, Fig. 3a also depicts the impact of our priming protocol to further increase Pfkfb3 expression in D2.A1 cells. The association of Pfkfb3 with metastatic competency prompted us to investigate the relationship between Pfkfb3 expression and breast cancer development. As such, we examined Pfkfb3 expression in several isogenic derivatives of the murine 4T1 progression series, including weakly tumorigenic 67NR cells, dormant and systematically invasive 4T07 cells, and fully metastatic 4T1 cells^31^. As expected, Pfkfb3 expression correlated with the aggressiveness of the 4T1 progression series (Fig. 3c). Likewise, a corresponding increase of Pfkfb3 expression was also observed in NMuMG cells transformed by overexpression of human EGFR (NME^32^) as compared with parental NMuMG cells (Fig. 3d). Finally, we also examined Pfkfb3 expression in a cohort of patient-derived xenograft (PDX) models comprising five basal breast cancer tumors and two HER2-enriched tumors (Fig. 3e, Supplementary Table 1)^33^. As expected, PDX tissues exhibited increased Pfkfb3 expression as compared to normal human mammary epithelial cells (HMECs). Similar elevations in Pfkfb3 mRNA (Fig. 3f) and protein (Fig. 3g) expression were observed in a variety of established human breast cancer cell lines relative to HMECs; however, it should be noted that the correlation between Pfkfb3 mRNA and protein expression varies slightly within human breast cancer cell lines. In light of these findings, we next sought to determine whether aberrant Pfkfb3 expression impacts RFS among a cohort of breast cancer patients using the Kaplan–Meier Plotter database (https://www.kmplot/analysis.com). Figure 3h shows that high Pfkfb3 expression correlated with reduced RFS in both basal/TNBC and HER2 enriched breast cancer subtypes, but not in their luminal A or B counterparts^34^. Collectively, these findings indicate that aberrant Pfkfb3 expression occurs frequently in metastatically proficient and aggressive breast cancers, leading to reduced RFS in breast cancer patients.

### Pfkfb3 expression drives emergence from metastatic dormancy

**Fig. 4:**
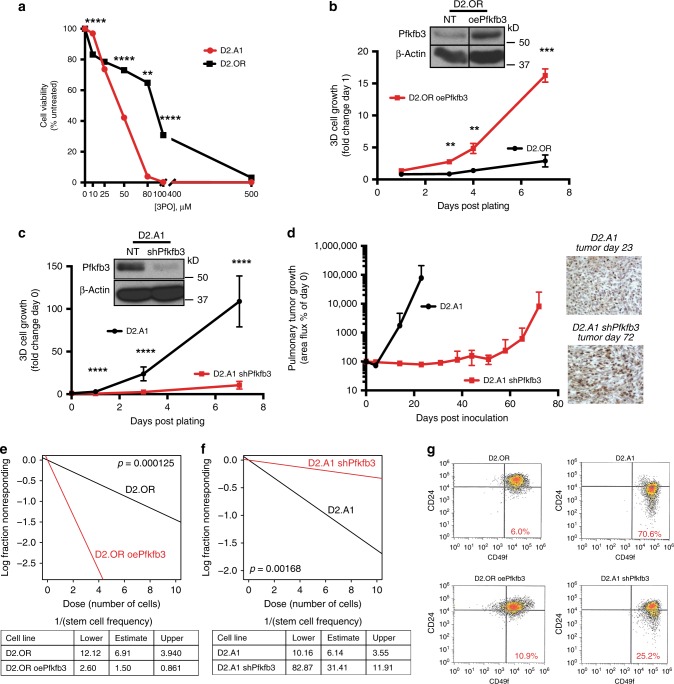
Aberrant Pfkfb3 expression drives emergence from metastatic dormancy and increased BCSC frequency. **a** D2.OR and D2.A1 cells were incubated in the absence or presence of the Pfkfb3 inhibitor, 3PO (0–500 μM), and differences in cell viability were quantified by CellTiter-Glo Assay. Data are the mean (±SD; *n* = 3). **P ≤ 0.01, ***P ≤ 0.001, and ****P ≤ 0.0001. This experiment was independently repeated for a total of three experiments, all with similar results. **b** Pfkfb3 overexpression drives D2.OR organoid growth in 3D cultures, while Pfkfb3-deficiency suppresses the growth of D2.A1 organoids in 3D culture, **c**, **d** Parental and Pfkfb3-deficient D2.A1 cells (1 × 10^6^ cells/injection) were inoculated into the lateral tail veins of BALB/c mice (*n* = 5). Pulmonary tumor growth was monitored by BLI and normalized to Day 0 readings. IHC images (×20 magnification) show loss of Pfkfb3 knockdown in tumors excised on Day 72. **e** Parental and Pfkfb3-overexpressing D2.OR cells or **f** parental and Pfkfb3-deficient D2.A1 cells were monitored for mammosphere forming ability by ELDA (*n* = 8). Stem cell frequency was evaluated using the ELDA: Extreme Limiting Dilution Analysis (http://bioinf.wehi.edu.au/software/elda/). These experiments were independently repeated for a total of three experiments, all with similar results. **g** Evaluating CD49f/CD24 expression levels by flow cytometry in parental and Pfkfb3-overexpressing D2.OR cells and in parental and Pfkfb3-deficient D2.A1 cells

Given the strong association of Pfkfb3 expression with breast cancer metastasis and aggressive disease states, we next sought to determine the impact of manipulating the expression and activity of Pfkfb3 on the outgrowth proficiency of D2-HAN derivatives. In doing so, we initially inhibited Pfkfb3 activity using the small-molecule Pfkfb3 inhibitor, 3PO^20^. As expected, 3PO administration was significantly more effective at killing metastatic D2.A1 cells (IC_50_ ~ 40 μM), which express robust levels of Pfkfb3 (Fig. 3) as compared to dormant D2.OR cells (IC_50 _~ 150 μM), which express negligible levels of Pfkfb3 (Fig. 4a). We also stably overexpressed Pfkfb3 (oePfkfb3) in dormant D2.OR cells (Fig. 4b, inset), which induced the robust outgrowth of D2.OR organoids in 3D cultures (Fig. 4b). In contrast, D2.A1 cells were rendered deficient in Pfkfb3 expression by transducing them with shRNA against Pfkfb3 (Fig. 4c, inset). Figure 4c shows that Pfkfb3-deficiency dramatically reduced the longitudinal growth of D2.A1 organoids, suggesting that Pfkfb3 expression is required for the outgrowth of metastatic cells in 3D cultures. Along these lines, we inoculated parental and Pfkfb3-deficient D2.A1 cells (D2.A1 shPfkfb3) into the lateral tail veins of BALB/c mice and found that reductions in Pfkfb3 expression significantly delayed the outgrowth of D2.A1 pulmonary tumors (Fig. 4d). Although depletion of Pfkfb3 expression did not wholly prevent the metastatic outgrowth of D2.A1 cells, it should be noted that the metastases that arose had in fact lost expression of the shRNA against Pfkfb3, as evidenced by the restoration of Pfkfb3 expression in late-developing D2.A1 pulmonary tumors (Fig. 4d, right panels). This result indicates that D2.A1 cells that possessed low levels of Pfkfb3 were negatively selected during the development and outgrowth of D1.A1 tumors in the lungs of mice. Collectively, these data suggest that metastatic dormancy is associated with low levels of Pfkfb3 expression, while emergence from metastatic dormancy and disease recurrence reflect high levels of Pfkfb3 expression.

Finally, BCSCs represent the tumor-initiating component of dormant breast cancer micrometastases, and as such, we hypothesized that Pfkfb3 could play an important role in mediating the stemness of these specialized breast cancer cells. To test this hypothesis, we performed limiting dilution mammosphere assays to evaluate the BCSC frequency in parental and Pfkfb3-manipulated D2-HAN derivatives. Figure 4e shows that overexpression of Pfkfb3 in D2.OR cells (D2.OR oePfkfb3) significantly increased their frequency of BCSCs as compared to parental D2.OR cells. Similarly, rendering D2.A1 cells deficient in Pfkfb3 (D2.A1 shPfkfb3) significantly reduced the frequency of their BCSCs (Fig. 4f). As a complementary measure of BCSC frequency, we showed in Fig. 4g that elevating Pfkfb3 expression in dormant D2.OR cells enhanced their acquisition of a CD49f^High^/CD24^Low^ phenotype, and conversely, depleting Pfkfb3 expression in metastatic D2.A1 cells suppressed their maintenance of a CD49f^High^/CD24^Low^ phenotype^35,36^. Taken together, these findings suggest that Pfkfb3 expression is critical for maintaining stemness within the BCSC compartment.

### Pfkfb3 expression is inversely related to autophagy

**Fig. 5:**
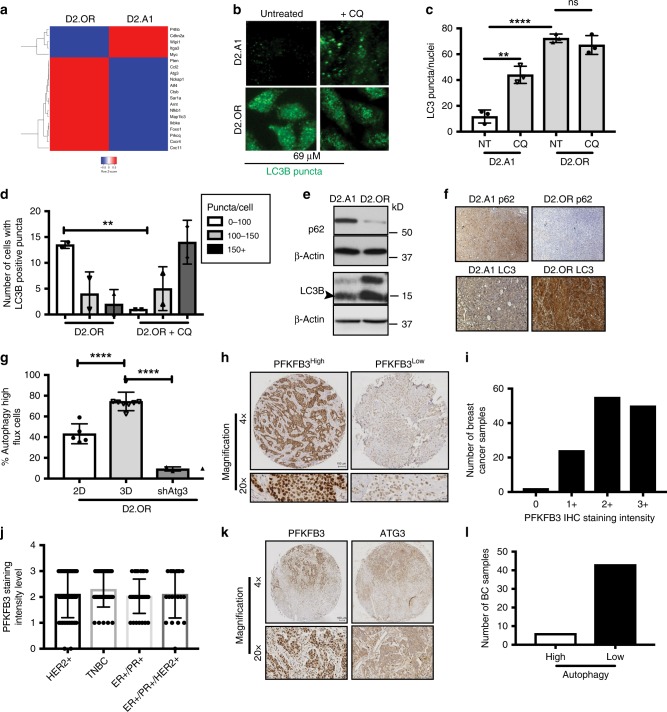
Pfkfb3 expression is inversely related to the levels of autophagy in breast cancer cells. **a** Dormant D2.OR cells derived from Fig. 1b express significantly more autophagy-associated as compared to their metastatic D2.A1 counterparts. Data are gene expression levels in D2.A1 cells normalized to D2.OR cells using a two-fold cutoff (http://www.heatmapper.ca/expression/). LC3B puncta accumulation (scale bar = 69 μM) (**b**) were quantified (**c**, **d**) using ImageJ particle analysis. D2.OR cells are more autophagic as compared to D2.A1 cells as determined by immunoblotting cell lysates (**e**) and IHC from mammary fat pad tumors (×10 magnification) harvested from Fig. 1e (**f**) for p62 and LC3B, and by autophagic flux using mCherry-GFP-LC3-expressing D2.OR cells propagated in 2D- or 3D cultures (**g**; data are the mean ± SEM; *n* = 4 for 2D-cultures; *n* = 6 for 3D cultures, ****P ≤ 0.0001). **h** One hundred and thirty breast cancer tumor slices were stained for PFKFB3 expression. Representative IHC images of PFKFB3 staining in (left) ER+/PR+/HER2+ and (right) ER+/PR+/HER2+ breast cancer samples. Scale bar (×4 and ×20 magnifications) = 100 μM. **i** In all, 80% (104/130) cores exhibit at least moderate (PFKFB3 intensity >2+) PFKFB3 staining and >98% (128/130) cores are PFKFB3 positive. **j** PFKFB3 staining intensity scores by breast cancer subtype. **k** Representative images of high PFKFB3 and low ATG3 IHC staining. **l** Immunohistochemical staining of breast cancer tumor cells with high PFKFB3 expression (3+ staining intensity; *n* = 49 samples) were evaluated for autophagy expression. Scale bar (×4 and ×20 magnifications) = 100 μM

In metastatic microenvironments, CSCs have been shown to upregulate autophagy pathways as a mechanism of cell survival^37^. As such, we hypothesized that high levels of autophagy may function in reducing Pfkfb3 expression, thereby promoting BCSC dormancy. Given the propensity for dormant D2.OR cells to colonize distant metastatic sites and remain quiescent for long periods of time, we investigated whether autophagy-based gene signatures were present in micrometastatic lesions derived from D2.OR cells (Fig. 2a). In accordance with our hypothesis, dormant D2.OR cells exhibited dramatically higher expression of autophagy-associated genes as compared to their metastatic D2.A1 counterparts (Fig. 5a). We confirmed the differential autophagy status between D2.OR (i.e., Autophagy^High^) and D2.A1 (i.e., Autophagy^Low^) cells via several independent analyses. First, we incubated D2.OR and D2.A1 cells in the absence or presence of the autophagy inhibitor, chloroquine (CQ), and subsequently monitored the formation of microtuble-associated protein 1A/1B-lightchain 3 (LC3B) puncta by immunofluorescent staining. As shown in Fig. 5b, c, metastatic D2.A1 cells exhibited significantly less LC3B puncta in both the absence and presence of CQ as compared to their dormant D2.OR counterparts. Although CQ appeared to have little impact on LC3B puncta formation detected across the entire population of D2.OR cells (Fig. 5c), we nonetheless observed CQ to induce a significant increase in LC3B puncta formation across subpopulations of D2.OR cells that possessed low (i.e., 0–100 puncta/cell), medium (100–150 puncta/cell), and high (>150 puncta/cell (Fig. 5d)). Thus, these findings are consistent with the notion that D2.OR cells are Autophagy^High^ and D2.A1 cells are Autophagy^Low^. This designation was reinforced by immunoblot analyses, which revealed that dormant D2.OR cells (i.e., Pfkfb3^Low^) exhibited elevated levels of LC3B and reduced levels of p62/SQSTM1 (Fig. 5e), a phenomenon that could be further modulated by administration of CQ (Supplementary Fig. 3a). Conversely, metastatic D2.A1 cells (i.e., Pfkfb3^High^) exhibited an inverse relationship between the expression of these same autophagy markers, a finding consistent with their designation as being Autophagy^Low^ (Fig. 5e). Thus, dormant D2.OR cells exhibit a Pfkfb3^Low^Authophagy^High^ phenotype that gives way to a Pfkfb3^High^Autophagy^Low^ phenotype in their metastatic D2.A1 counterparts, a relationship that also occurred in tumors produced by D2.A1 and D2.OR cells (Fig. 5f). This notion was reinforced by monitoring alterations in autophagic flux using the pH-sensitive mCherry-GFP-LC3 reporter^29,38^. Indeed, Fig. 5g shows that introducing mCherry-GFP-LC3-expressing D2.OR cells into 3D cultures readily induced autophagic flux as evidenced by an increase in mCherry:GFP ratio as compared to their 2D-cultured counterparts (Fig. 5g, Supplementary Fig. 3b). Importantly, inactivating autophagy by rendering D2.OR cells deficient in Atg3 expression dramatically reduced their ability to elicit autophagic flux (Fig. 5g). Finally, immunofluorescent analyses showed increased colocalization of LC3B and LAMP1 (Supplementary Fig. 3c), which serves as a marker for enhanced formation of autophagolysosomes in autophagic cells. Collectively, these findings suggest that Pfkfb3 expression is inversely related to the activation status of autophagy, such that dormant D2.OR cells exhibit a Pfkfb3^Low^Autophagy^High^ phenotype, while their metastatic D2.A1 counterparts exhibit a Pfkfb3^High^Autophagy^Low^ phenotype.

To extend the relevance of these findings to human breast cancers, we utilized a breast tissue microarray (TMA) to evaluate Pfkfb3 expression by immunohistochemistry (IHC) across the four major clinical breast cancer subtypes: (i) ER^−^/PR^−^/HER2^−^ (TNBC); (ii) ER^+^/PR^+^/HER2^−^ (luminal A); (iii) ER^+^/PR^+^/HER2^+^ (luminal B); and (iv) ER^−^/PR−/HER2^+^ (HER2+)^39^. Figure 5h depicts representative Pfkfb3 staining, while Fig. 5i shows that greater than 98% of tumor cores were positive for Pfkfb3 expression, with 80% (104/130 cores) exhibiting a Pfkfb3 intensity score of at least 2^+^ (i.e., moderate staining). Furthermore, we observed Pfkfb3 staining intensity to be varied both within and amongst individual breast cancer subtypes (Fig. 5j), thereby demonstrating that Pfkfb3 expression is frequently upregulated across the spectrum clinical breast cancer subtypes. Finally, we investigated the autophagic state of these same human breast cancer tissues by IHC staining for the autophagy mediators p62/SQSTM1 (Supplementary Fig. 4a, b), LC3 (Supplementary Fig. 4c, d), and Atg3 (Supplementary Fig. 4e, f), whose expression was compared with that of Pfkfb3 (Fig. 5k). Autophagy is a dynamic process that can be difficult to assess in fixed human tissues, which represent only a static snapshot of the pathophysiology associated with primary human tumors. Given these limitations, we nevertheless found that 94% (46/49) of breast tumors that exhibited high Pfkfb3 expression (i.e., 3^+^ staining intensity) also displayed low levels of autophagy (Fig. 5l). Collectively, these findings suggest that metastatic dormancy reflects the acquisition of a Pfkfb3^Low^Authophagy^High^ phenotype that transitions to a Pfkfb3^High^Autophagy^Low^ phenotype during metastatic relapse.

### Autophagy inactivation induces metastatic dormancy emergence

**Fig. 6:**
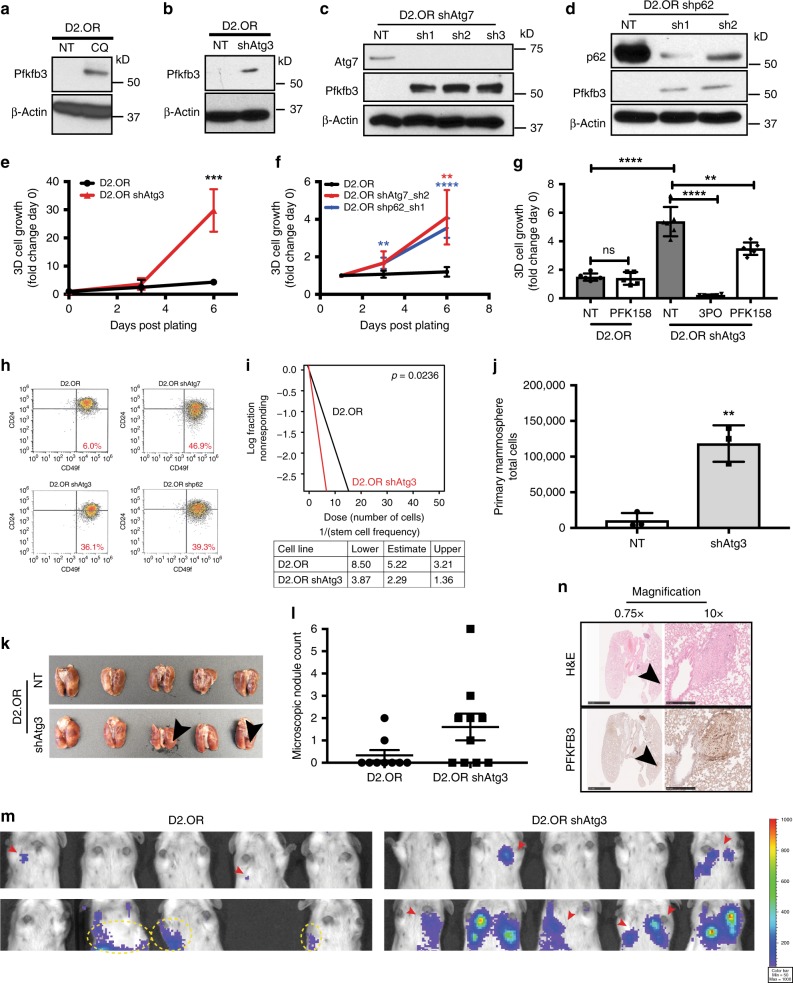
Inactivation of autophagy induces BCSC expression of Pfkfb3 and emergence from metastatic dormancy. Inactivation of autophagy induces Pfkfb3 expression in D2.OR cells. Autophagy was inhibited by administration of chloroquine (CQ; 25 μM; **a**), and by shRNA-mediated depletion of either Atg3 (**b**), Atg7 (**c**), or p62 (**d**). Inactivation of either Atg3 (**e**, ***P ≤ 0.001), Atg7 (**f**, **P ≤ 0.01), or p62 (**f**, **P ≤ 0.01; ****P ≤ 0.0001) promotes D2.OR organoid outgrowth in 3D cultures. This experiment was independently repeated for a total of 3 experiments, all with similar results. **g** Dual inhibition of autophagy through Atg3 knockdown and Pfkfb3 by PFK-158 treatment inhibits D2.OR organoid outgrowth in 3D cultures. Data represent mean (±SD) of biological replicates performed in triplicate (ns *P* > 0.05, **P ≤ 0.01, and ****P ≤ 0.0001). **h** CD49f/CD24 flow cytometry evaluation in parental and Atg3-deficient D2.OR cells. **i** BCSC frequency as determined by an ELDA mammosphere assay. **j** Quantification of total cell numbers isolated following completion of the primary mammosphere assays depicted in **i**. Data demonstrate significantly larger spheres associated with Atg3-deficient D2.OR cells as compared with parental D2.OR cells. **k** Parental (i.e., NT) and Atg3-deficient D2.OR cells were inoculated into the lateral tail veins of BALB/c mice (2 × 10^6^ cells/mouse) and pulmonary tumor development was monitored by BLI. Photomicrograph shows macroscopic tumors (black arrows) in 2/5 mice inoculated with Atg3-deficient D2.OR cells. **l** Microscopic nodule count from lung tumors in **k**. **m** Bioluminescence imaging (BLI) of Day 70 D2.OR and D2.OR shAtg3-derived tumors. Red arrows indicate the presence of tumor. Yellow circles indicate BLI signal that does not derive from lung tumors, but instead from nonspecific signals present in tail tumors. A scale bar is included where red represents the maximum of the figure (1000) while violet represents the minimum (50). **n** Representative IHC images show elevated Pfkfb3 expression in tumor nodules produced by Atg3-deficient D2.OR cells. Scale bar (×0.75 magnification) = 2.5 mm. Scale bar (×10 magnification) = 250 μM

Because emergence from dormancy reflects a phenotypic transition from a Pfkfb3^Low^Authophagy^High^ state to a Pfkfb3^High^Autophagy^Low^ state, we hypothesized that inactivating autophagy through either pharmacologic (e.g., CQ; Supplementary Fig. 5a; see refs. ^40,41^) or genetic means would elicit upregulated expression of Pfkfb3, resulting in D2.OR cell outgrowth in 3D cultures. As anticipated, acute CQ administration (3 h) to inhibit of autophagy resulted in the robust expression of Pfkfb3 in D2.OR cells (Fig. 6a). Unfortunately, we were unable to monitor the impact of chronic CQ administration (5–7 days) on Pfkfb3 expression in D2.OR organoids due to its cytotoxic activities, which were surprisingly similar across several human and murine breast cancer cell lines (Supplementary Fig. 5b–d). Although by no means definitive, these findings imply that chronic application of CQ killed cells through autophagy-dependent and -independent mechanisms.

To circumvent the difficulties associated with CQ administration, we genetically inhibited autophagy and monitored the resultant outgrowth in 3D cultures. Accordingly, rendering D2.OR cells deficient in Atg3 expression (Supplementary Fig. 6a) elicited dramatic upregulation of Pfkfb3 expression in dormant D2.OR cells (Fig. 6b), as did depleting the expression of Atg7 (Fig. 6c, Supplementary Fig. 6b), p62/SQSTM1 (Fig. 6d, Supplementary Fig. 6c), or FIP200 (Supplementary Fig. 6d). More importantly, we found that knockdown of Atg3 in dormant D2.OR cells promoted their outgrowth in 3D cultures (Fig. 6e), as did rendering these cells deficient in the expression of additional autophagy mediators, including Atg7 (Fig. 6f), p62/SQSTM1 (Fig. 6f), and FIP200 (Supplementary Fig. 6e). Thus, measures capable of inactivating autophagy appear to be competent in promoting Pfkfb3 expression and the reactivation of proliferative programs in dormant breast cancer cells. A corollary states that the proliferation of Pfkfb3^High^Autophagy^Low^ D2.OR cells engendered by genetic depletion of Atg3 (Supplementary Fig. 6a) should be neutralized by pharmacologic inactivation of Pfkfb3. Accordingly, Fig. 6g shows that the growth of Atg3-deficient D2.OR organoids was significantly reduced by administration of the Pfkfb3 inhibitors, 3PO (Fig. 4) or PKF158^20^. Moreover, Atg3-deficient D2.OR cells, which exhibit upregulated expression of Pfkfb3 (Fig. 6b), were significantly more sensitive to administration of PFK-158, thereby reducing their overall viability in 2D-cultures (Supplementary Fig. 6f).

Given the importance of BCSCs on tumor outgrowth, we next evaluated the impact of autophagy inactivation on the stemness of D2.OR cells. As shown in Fig. 6h, autophagy-deficient D2.OR cells (i.e., those lacking expression of Atg3, Atg7, or p62/SQSTM1) demonstrated robust acquisition of a CD49f^High^/CD24^Low^ phenotype that is consistent with increased stem cell frequency elicited by autophagy inactivation. Similar enhancement in stemness was noted in mammosphere ELDA (Fig. 6i), and in the relative size of the resultant mammospheres (Fig. 6j). Finally, we extended these analyses to monitor the impact of Atg3-deficiency on the outgrowth proficiency of D2.OR cells in the lungs of BALB/c mice. As compared to their parental counterparts, D2.OR cells rendered deficient in Atg3 expression acquired the capacity to form pulmonary tumors (Fig. 6k–m), which exhibited robust Pfkfb3 expression (Fig. 6n). Collectively, these findings reinforce the notion that emergence from metastatic dormancy reflects a loss of autophagy-associated Pfkfb3^Low^Autophagy^High^ phenotypes to a gain of Pfkfb3-associated Pfkfb3^High^Autophagy^Low^ phenotypes.

### Pfkfb3 is an autophagy substrate that binds to p62/SQSTM1

**Fig. 7:**
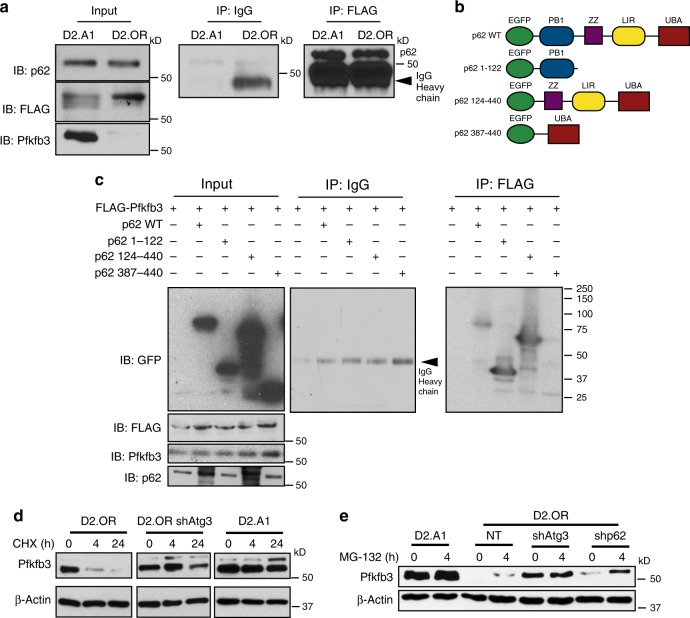
Pfkfb3 is an autophagy substrate that binds to the autophagosome cargo protein p62/SQSTM1. **a** FLAG-tagged Pfkfb3 was transiently expressed in D2.A1 and D2.OR cells and captured by immunoprecipitation with anti-FLAG antibodies. Afterward, p62/SQSTM1 and Pfkfb3 were visualized by immunoblotting as indicated. **b** Schematic of wild type and p62/SQSTM1 truncation mutants. **c** Immunoblot (IB) analysis of FLAG-tagged Pfkfb interaction with wild type and mutant p62/SQSTM1 constructs depicted in panel **b**. **d** Cycloheximide (CHX) [25 μM] was administered for 0–24 h as indicated. Pfkfb3 was stabilized in Autophagy^Low^ (i.e., D2.A1 and D2.OR shAtg3) cells but not in Autophagy^High^ (i.e., D2.OR) cells. **e** MG132 [10 μM] treated for 0–4 h as indicated. Pfkfb3 expression in D2.A1, D2.OR, D2.OR shAtg3, and D2.OR shp62 was evaluated by immunoblotting

Given the propensity for autophagy to maintain dormant states and suppress Pfkfb3 expression, we reasoned that Pfkfb3 expression may be governed by direct interaction with autophagy machinery, specifically the cargo adaptor protein, p62/SQSTM1 (refs. ^7,14^). Structurally, p62/SQSTM1 comprises six functional domains, of which the best characterized are: (i) Phox/Bem1 (PB1) domain; (ii) zing-finger domain (ZZ); (iii) LC3-interacting region (LIR); and (iv) ubiquitin-associated domain (UBA)^42^. The accumulation of p62/SQSTM1 enables its binding to target proteins through two major mechanisms. First, the UBA domain of p62/SQSTM1 can aggregate and sequester proteins, thereby preventing their proteasomal degradation. Second, the LIR domain of p62/SQSTM1 can target proteins for autophagic degradation^42^. To determine whether p62/SQSTM1 interacts with and stabilizes the expression of Pfkfb3, D2.OR and D2.A1 cells were transiently transfected with FLAG-tagged Pfkfb3, which was captured by immunoprecipitation with anti-FLAG antibodies. As shown in Fig. 7a, endogenous p62/SQSTM1 was readily detected in anti-FLAG immunocomplexes, suggesting that Pfkfb3 interacts physically with p62/SQSTM1. Interestingly, when human HEK-293 cells were transiently transfected with p62/SQSTM1 fused to GFP (pDestEGFP^43^; Supplementary Fig. 7a, b), we observed a robust induction of Pfkfb3 protein (Supplementary Fig. 7b), a finding consistent with a reduction in cellular autophagy elicited by elevated p62/SQSTM1 expression. Moreover, recombinant p62/SQSTM1 readily bound to polyubiquitinated Pfkfb3 (Supplementary Fig. 7c), suggesting that Pfkfb3 interacts with the UBA domain of p62/SQSTM1 (ref. ^44^). Structure–function analyses were undertaken to map the domain(s) in p62/SQSTM1 responsible for binding Pfkfb3 (Fig. 7b). As shown in Fig. 7c, Pfkfb3 interacted robustly with p62/SQSTM1 constructs that contained its PB1 and UBA domains, events that appear to function in enhancing the stability of Pfkfb3. In support of this notion, we performed experiments to evaluate the protein stability and proteasomal degradation of Pfkfb3 in both Pfkfb3^Low^/Autophagy^High^ (i.e., D2.OR) and Pfkfb3^High^/Autophagy^Low^ (i.e., shAtg3-D2.OR and D2.A1) cells. Inhibition of protein biosynthesis by administration of cycloheximide demonstrated that Pfkfb3^Low^/Autophagy^High^ cells rapidly turnover Pfkfb3 expression as compared to their Pfkfb3^High^/Autophagy^Low^ counterparts, which exhibit remarkable stability of Pfkfb3 (Fig. 7d). Furthermore, proteasome inhibition in response to MG132 treatment readily promoted the stability of Pfkfb3 in Pfkfb3^Low^/Autophagy^High^ cells (Fig. 7e). Importantly, inhibition of the proteasome in p62/SQSTM1-deficient cells stabilized Pfkfb3 expression to levels approaching those detected in autophagy low D2.A1 cells. Finally, it is important to note that the synthesis of Pfkfb3 transcripts was unaffected by autophagy modulation, indicating that upregulation of Pfkfb3 transpires post-translationally, not transcriptionally (Supplementary Fig. 7d). Collectively, these findings indicate that autophagy activation contributes to metastatic dormancy in part by facilitating p62/SQSTM1-mediated autophagic degradation of Pfkfb3. As such, our findings support the notion that pharmacologic and genetic inactivation of autophagy can contribute to emergence of metastatic dormancy and disease recurrence via Pfkfb3 stabilization and the reactivation of proliferative programs.

## Discussion

Following dissemination to distant metastatic sites, BCSCs can enter a dormant, non-proliferative state during which chemoresistance and immune evasion can render cells viable for years or even decades^6,7^. Both intrinsic and extrinsic factors can influence breast cancer dormancy, which if perturbed can result in the reinitiation of proliferative programs and escape from metastatic dormancy. Here we show that dormant cells are highly autophagic and express low levels of Pfkfb3; however, upon inhibition of autophagy, unbridled Pfkfb3 expression drives escape from metastatic dormancy. More importantly, our findings implicate Pfkfb3 as an autophagy substrate that interacts physically with the cargo adaptor protein, p62/SQSTM1, thereby modulating the expression levels of Pfkfb3 in disseminated breast cancer cells. Along these lines, genetic and epigenetic alterations in BCSCs, as well as in their niches can have a profound influence on the activation status of autophagy in dormant BCSCs, thus regulating the phenotypic shift between Pfkfb3^Low^Autophagy^High^ (i.e., dormancy) and Pfkfb3^High^Autophagy^Low^ (i.e., metastatic recurrence) profiles.

Upon disseminating to distant metastatic sites, CSCs must navigate hostile microenvironments and survive an onslaught of microenvironmental stressors, including hypoxia, nutrient deprivation, and mechanical stressors. Autophagy is a critical mechanism whereby DTCs adapt to nutrient stress induced by new metastatic microenvironments^3,45–47^. Our study bolsters recently published findings that autophagy is a critical component in maintaining dormant states^29^. However, our findings significantly extend this work by elucidating the dynamic interplay between Pfkfb3 and autophagy in governing metastatic outgrowth, an event initiated via autophagy-mediated targeting of Pfkfb3 expression. Importantly, we show that inhibition of autophagy through either pharmacologic or genetic means renders dormant cells competent to undergo metastatic outgrowth both in vitro and in vivo, a phenomenon dependent upon aberrant Pfkfb3 (Fig. 6). Future studies need to further explore the dynamics between Pfkfb3 mRNA and protein expression in the context of autophagy and metastatic dormancy, particularly since Pfkfb3 expression appears to be regulated in both a transcriptional and post-translational manner. Although beyond the scope of the current study, future investigations also need to elucidate the signals responsible for driving the production of Pfkfb3 transcripts in primed BCSCs.

Pfkfb3 was first identified as an essential mediator of glycolysis via its ability to induce cellular glycolytic metabolism^18,48^. In physiological settings of reduced nutrient availability that result in the activation of autophagic pathways, it is perhaps not surprising that the expression of genes coupled to metabolic activity (e.g., Pfkfb3) is suppressed. The relationship between Pfkfb3 and autophagy remains to be fully clarified in the scientific literature. For instance, pharmacologic or genetic inactivation of Pfkfb3 has been linked to the diminished capacity of AMPK to activate autophagy in human kidney cells^17^, while the loss of Pfkfb3 expression inhibited autophagy in CD4^+^ T cells isolated from patients with rheumatoid arthritis^15^. In contrast, inactivation of Pfkfb3 activity has also been shown to elicit the compensatory activation of autophagy, thereby enhancing the survival of colorectal cancer cells^16^. Our findings establish that breast cancer cells possess a dynamic degree of plasticity that enables them to readily switch between glycolytic and autophagic phenotypes (Fig. 6); they also show that simultaneous inactivation of both survival mechanisms may offer new inroads into eliminating dormant disseminated breast cancer cells, findings reminiscent of those employing dual targeting of Pfkfb3 and mitophagy/autophagy to alleviate mitotic-arrested breast cancer cells^49^. Collectively, these findings bolster our contention that monitoring the balance between Pfkfb3 and autophagy will offer important insights into the disease status and long-term outcomes of metastatic breast cancer patients.

Historically, basic and clinical research findings have highlighted the context-dependent role of autophagy in regulating tumorigenesis. Indeed, in the earliest stages of tumor growth and development, autophagy functions as a tumor suppressor, thereby limiting tumor growth. However, upon establishment of the primary tumor or distant metastasis, autophagy can promote tumor progression by subverting stress responses, and consequently, promoting tumor cell survival and disease progression^50^. Identifying the biological basis behind the switch in tumor cell response to autophagy will be a key step in developing autophagy modulating drugs and in maintaining BCSCs in a state of metastatic dormancy. Accordingly, numerous clinical trials have aimed to inhibit autophagy as a cancer therapeutic with varying degrees of success^50^. In stark contrast, our findings suggest that the targeted inactivation of autophagy could in fact render dormant DTCs highly proliferative, resulting in their ability to increase metastatic burden and ultimately tumor recurrence. The dual role of autophagy in tumor progression highlights the importance in identifying those tumors most likely to respond to autophagy inhibition as compared to those tumors likely to progress through a Pfkfb3-dependent mechanism. Along these lines, it should be noted that PFKFB3 expression failed to correlate with hormone receptor status, suggesting that autophagy may play a hormone-independent role in maintaining BCSC dormancy. Thus, in moving forward toward clinical settings, we suggest that subtype-specific combination therapies could prove to be highly effective in stymieing the ability of disseminated BCSCs to emerge from metastatic dormancy and recur.

Finally, while it is tempting to postulate Pfkfb3 as a sole driver in mediating escape from metastatic dormancy, the actions of one single gene are likely insufficient to modulate such a multifactorial event. As such, future studies need to identify upstream regulators that mediate Pfkfb3 gene expression, as well as those genes responsible for facilitating escape from metastatic dormancy. For example, we and others^51^ have observed transforming growth factor-β (TGF-β) to induce Pfkfb3 gene expression following activation of both its canonical and noncanonical effectors. Dormancy-associated gene signatures are highly redundant with those associated with epithelial–mesenchymal transition (EMT) programs, suggesting that dormant states are one facet among the spectrum of phenotypes attributed to EMT programs^30,32^. As such, future studies need to determine the impact of noncanonical TGF-β signaling systems in driving the acquisition of and eventual emergence from metastatic dormancy, as well as assess the role of Pfkfb3 in mediating these events. Our findings suggest that clinical monitoring of Pfkfb3 expression and autophagy status could provide for a means to stratify patient response to potential autophagy targeting strategies, as well as provide insights into long-term prognosis. Moreover, our findings also suggest that autophagy activation, not inhibition, could function to alleviate aberrant Pfkfb3 expression and maintain disseminated BCSCs in a state of perpetual dormancy, thus prolonging the overall survival of breast cancer patients.

## Methods

### Cell culture and reagents

Murine breast cancer cell lines from the D2.HAN series and 4T1 progression series were obtained from Fred Miller (Wayne State University, Detroit, MI) and grown in Dulbecco's modified Eagle's medium (DMEM) (Sigma Aldrich) supplemented with 10% fetal bovine serum (FBS) and 1% Pen/Strep. D2.OR cells engineered to stably express mCherry-GFP-LC3 reporter were described previously^29,38^. Stable expression of firefly luciferase in D2.HAN cells was achieved by transfection with pNifty-CMV-luciferase, followed by selection with zeocin (500 μg/mL; Invitrogen)^30^. Murine Pfkfb3 was functionally disrupted by transducing D2.A1 cells with lentiviral particles that encoded for shRNA against Pfkfb3 (Supplementary Table 2), followed by selection with puromycin (5 μg/mL). Similarly, murine autophagy components were functionally disrupted by transducing D2.OR cells with lentiviral particles that encoded for shRNA against either Atg3, Atg7, p62, or FIP200 (Supplementary Table 2), followed by selection with puromycin (5 μg/mL). Human breast cancer cell lines were obtained from ATCC and cultured as described in Supplementary Table 3. HMECs were obtained from the laboratory of Mark Jackson (Case Western Reserve University, Cleveland, OH)^52^.

The DNA fragment encoding murine Pfkfb3, pDONR223-PFKFB3, was obtained from William Hahn and David Root (Addgene plasmid # 23668)^53^. pDONR233-PFKFB3 was introduced into the pLenti-CMV GFP DEST and the pWZL-Neo-Myr-Flag-DEST (Addgene plasmid # 15300) vector by Gateway™ Cloning using the Gateway™ LR Clonase™ II Enzyme Mix (Invitrogen, 11791-020) by LR recombinase reaction. D2.OR cells were transduced with lentiviral particles that encoded for Pfkfb3 and stable polyclonal populations of Pfkfb3-expressing D2.OR cells were isolated by flow cytometry for GFP (Sony Reflection HAPS1 flow cytometry sorter). The Gateway expression vector pDestEGFP-p62 was constructed by Bjørkøy et al.^43^, while the ubiquitin cDNA (pcDNA-HA-ubiquitin) was a gift from Dr. Gerald Blobe (Duke University, Durham, NC). Both the pDestEGFP-p62 and the pcDNA-HA-ubiquitin were introduced into HEK-293 cells using the *Trans*IT®-LT1 Transfection Reagent (Mirus, MIR2300).

Cells used in experimental studies were passaged fewer than 50 times, although most cell lines used for the generation of the data herein were passaged fewer than 20 times prior to data acquisition. Cell lines tested negative for mycoplasma using the MycoAlert Mycoplasma Detection Kit (Lonza, LT07–218) throughout the study with the most recent negative mycoplasma test performed October 2018.

### Cell priming and mammosphere assays

D2.OR and D2.A1 cells were grown on Cultrex reconstituted basement membrane extracts (Trevigen, 3533-001-02) for 3–5 days and transferred to ultra-low attachment plates (Coaster, 3471) for mammosphere assays. Cells were harvested from Cultrex reconstituted basement membrane extracts using the Cultrex® 3D Culture 10X Cell Harvesting Buffer (Treviegen, 3448-020-01) according to the manufacturer’s instructions. Mammospheres were grown for ~10 days in DMEM/F12 media (Gibco-Thermo Fisher) supplemented with bGFG (20 ng/mL; Invitrogen), EGF (20 ng/mL; Invitrogen), B27 (Gibco, 17504), Pen/Strep (2.5 mL), and Heparin (4 μg/mL; Sigma Aldrich). Limiting dilution assays were performed by single cell sorting into ultra-low attachment plates using the Sony Reflection HAPS1 cell sorter. Assays were analyzed using the Extreme Limiting Dilution Analysis (ELDA) software^54^ at various timepoints throughout 2017 and 2018.

### Animal studies

To monitor changes in pulmonary outgrowth, luciferase-expressing parental D2.HAN cells and their Pfkfb3-deficient D2.A1 (1 × 10^6^ cells/mouse) and Atg3-deficient D2.OR counterparts (2 × 10^6^ cells/mouse) were inoculated into the lateral tail vein female BALB/c mice (4–5 weeks old; JAX), at which point pulmonary tumor development was monitored biweekly by bioluminescent imaging (BLI) on an IVIS Spectrum In Vivo Imaging System (Perkin Elmer)^11^. Additionally, alterations in BCSC frequency between D2.OR and D2.A1 cells was assessed by implanting these cells into the mammary fat pads of female BALB/c mice (4–5 weeks old; JAX) at varying concentrations (100–50,000 cells/mouse). Tumor latency and subsequent development was monitored weekly using digital calipers (Fisher Scientific) and tumor volume was calculated using the formula: volume = (width^2^) × (length/2). Estimated stem cell frequencies based on the absence or presence of mammary tumors were generated at a 95% confidence interval using http://bioinf.wehi.edu.au/software/elda/.

All animal experimental protocols were approved by the Institutional Animal Care and Use Committee of Case Western Reserve University, and all animal studies herein were performed in accordance with these approved protocols. We complied with all relevant ethical regulations for animal testing and research. This study was conducted under the Protocol # 2013-1020 entitled “Role of TGFβ-regulated Proteins in Breast Cancer Growth and Metastasis.”

### Laser capture microdissection and microarray analysis

Formalin-fixed, paraffin-embedded (FFPE) sections of lung tumor samples were stained with crystal violet to visualize tumor morphology. Afterward, a Leica LMD 7000 Laser Microdissection System was utilized to isolate spindle and epithelioid tumor nodules. Total RNA extraction was accomplished using the RNeasy FFPE Kit (Qiagen, 73504), at which point the Affymetrix Sensation Plus labeling protocol was used to prepare the RNA samples for interrogation on Affymetrix GeneChip Mouse 2.0 ST microarrays. Probe hybridization was performed according to the manufacturer’s recommendations, and the resulting probe signals were normalized using the Affymetrix Expression Console Software package. Data analysis was performed using the Affymetrix Transcriptome Analysis Console, Ingenuity Pathway Analysis, and KEGG analysis software.

### Real-time PCR

Total RNA was isolated using the RNeasy Plus Mini Kit (Qiagen, 74134). The nucleic acid purity and concentration was examined using a NanoDrop Spectrophotometer. Subsequently, purified total RNA (1 μg/reaction) was reverse transcribed with the iScript™ cDNA Synthesis Kit (Bio-Rad, 1708891) and the resulting cDNA was utilized for semi-quantitative real-time PCR using the iQ SYBR Green Supermix (Bio-Rad, 170-8880) using the oligonucleotide primer pairs as described in Supplementary Table 4.

### Immunofluorescence

D2.A1 and D2.OR cells were allowed to adhere overnight to glass coverslips in a 12-well plate. The following morning, CQ (25 μM/well) was added for 4 h, at which point the cells were fixed in 4% paraformaldehyde, permeabilized in 0.1% Triton X-100, and stained with anti-LC3B antibody at a 1:250 dilution (Abcam, ab225383) for 1 h at room temperature. In some experiments, LC3B puncta were visualized using a Lecia DM6000 fluorescent microscope and the Analyze Particles plugin in ImageJ was used for particle quantification. Alternatively, the formation of autophagolysosomes was evaluated by monitoring LC3B and LAMP1 immunofluorescence using a Keyence BZ-X800 and the Haze Reduction feature.

### Immunoblotting

Cells were harvested on ice in RIPA lysis buffer (50 mM Tris, HCl, pH 7.4, 150 mM sodium chloride, 0.25% sodium deoxycholate, 1% IGEPAL-630, 0.1% SDS, 1 mM protease inhibitor cocktail (CalBiochem), 10 mM sodium ortho-vanadate, 40 mM β-glycerophosphate, and 20 mM sodium fluoride). Lysates were clarified by microcentrifugation and the resulting supernatants were immunoblotted using the antibodies listed in Supplementary Table 5 (ref. ^55^) (see Uncropped gel images, Supplementary Fig. 9).

### Co-immunoprecipitation

For co-immunoprecipitation assays, the cells were transiently transfected either singly or in multiple combinations with recombinant GFP-tagged p62/SQSTM1 (pDestEGFP-p62 (ref. ^43^)), FLAG-tagged Pfkfb3 (pWZL-Neo-Myr-Flag-DEST Addgene plasmid # 15300), or HA-tagged ubiquitin (pcDNA-HA-ubiquitin). After 48 h, the cells were lysed and solubilized on in Buffer H (50 mM β-glycerophosphate, 150 mM sodium chloride, 1.5 mM ethylenediaminetetraacetic acid, 1 mM dithiothreitol, 0.1 mM sodium ortho-vanadate, and 1% Triton X-100), and subsequently were sonicated three times (10 s pulses at 35% amplitude) before being clarified in a microcentrifuge (12,000 r.p.m. for 10 min at 4 °C). Afterward, 50 μL of sample was removed as input, with the remaining lysate being subjected to overnight immunoprecipitation using anti-FLAG antibodies (Sigma, F3165) and protein A and protein G beads (GE Healthcare 17-5280-01 and 17-0618-01, respectively). Immunocomplexes were collected by centrifugation and washed three times before fractionation through 10% SDS-PAGE gels. Following electrophorectic transfer to nitrocellulose, captured proteins were visualized by immunoblotting as above (see Uncropped gel images, Supplementary Fig. 9).

### Cell biological assays

Alterations in cell viability elicited by administration of varying concentrations of the Pfkfb3 inhibitors 3PO (0–500 μM) and PFK-158 (0–500 μM) was determined using The CellTiter-Glo® Luminescent Cell Viability Assay (Promega, G7570) according to the manufacturer’s instructions. Additionally, differences in 3D-longitudinal growth were determined by culturing luciferase-expressing parental D2.OR and D2.A1 cells and their derivatives (2000 cells/well) in 96-well plates (Costar, 3610) on a bed of solidified Cultrex (50 μL) reconstituted basement membrane extract (Trevigen, 3533-001-02) in DMEM media supplemented with 10% FBS and 5% Cultrex^10^. Luminescence was measured by adding D-luciferin potassium salt (Gold Biotechnology, LUCK-100) to each well and the signal was read using a GloMax-Multi detection system (Promega). Initial readings were used to normalize subsequent bioluminescent readings to measure longitudinal 3D growth.

### Immunohistochemistry

Lung and tumor tissue were removed during necropsy and immediately placed in 10% neutral buffered formalin for 24 h prior to processing and paraffin embedding. Five-micron sections were cut and slides were used within 2 weeks of sectioning. Immunohistochemistry for p62, LC3, Pfkfb3, and Atg3 was performed using the Leica Novolink Polymer Kit (Leica Biosystems, RE7200-CE) according to the manufacturer’s instructions; however, the Post-Primary reagent was omitted for rabbit primary antibodies as described in Supplementary Table 5.

Immunohistochemistry staining was evaluated by a Board-certified pathologist (B.C.C). Briefly, the intensity of staining was evaluated on a scale from 0 to 3 where 0 indicated the absence of positive staining, 1 indicated light positive staining, 2 indicated moderate staining, and 3 indicated strong staining. The percentage of cells at each staining intensity (0–3) was evaluated. The uniformity of staining was designated as either non-homogeneous where <100% of positive tumor cells exhibited staining at a given intensity level or homogeneous where 100% of cells stained positive at the given intensity level.

We complied with all relevant ethical regulations for the work with human participants and completed human subjects training through the CITI Program. The human breast cancer TMAs were obtained from the Case Comprehensive Cancer Center in accordance with the following approved IRB protocols: CASE 01-13-43 C and CASE 7114. Access to and approved use of the TMAs occurred under the Tissue Research and Review Committee protocol #TRRC1118 entitled “Pfkfb3 Modulates Breast Cancer Stem Cell Metastatic Latency and Recurrence Through Autophagy.

### CD49f/CD24 flow cytometry

Cells were harvested from adherent plates using Accutase™ Cell Detachment Solution (BD, 561527) for 10 min at 37 °C. Accutase was inactivated using DMEM with 10% FBS and cell pellets were washed with PBS. Cells were resuspended in FACS buffer (1× PBS and 0.5% BSA), passed through 35 μm cell strainers (Falcon, 352235), and incubated with anti-CD49f PE (Invitrogen, 12-0495-82) and -CD24 (BD, 562563) antibodies for 1 h in the dark according to the manufacturer’s recommendations. Afterward, the cells were washed three times with 1× PBS and resuspended 1 mL of FACs buffer immediately prior to analysis on an Attune NxT flow cytometer (see Gating Strategy, Supplementary Fig. 8) (Invitrogen).

### Autophagy flux flow cytometry

D2.OR cells that stably expressed the autophagy flux reporter, mCherry-GFP-LC3 (ref. ^29^), were analyzed on an Attune NxT flow cytometer (Invitrogen). Autophagic flux was determined by analyzing the ratio of mCherry to GFP in mCherry-GFP-LC3-expressing D2.OR cells. Cells that exhibited mCherry:GFP ratios of ≥1 were designated as being Autophagy^High^ ^29^.

### Statistical analyses

Limiting dilution mammosphere assays and in vitro stem cell frequency analyses were performed using the ELDA software to test for stem cell frequency differences between groups^55^. Unless otherwise stated, all experiments were performed using biological triplicates where significance was determined by unpaired two-tailed Student’s *T*-tests. *P* values ≤0.05 were considered to be statistically significant and were denoted as follows: *P ≤ 0.05; **P ≤ 0.01; ***P ≤ 0.001; and ****P ≤ 0.0001.

### Reporting Summary

Further information on research design is available in the Nature Research Reporting Summary linked to this article.

## Supplementary information

Supplementary Information

Reporting Summary

## Data Availability

The data generated in this study are included in the published article and in the Supplementary Information and are available from the authors on reasonable request. The microarray data were deposited to the GEO Database under the accession number: GSE131890.

## References

[1] American Cancer Society. Cancer facts & figures 2015 (ACS, Atlanta, 2015).

[2] O’Shaughnessy J (2005). Extending survival with chemotherapy in metastatic breast cancer. Oncologist.

[3] Sosa MS, Bragado P, Aguirre-Ghiso JA (2014). Mechanisms of disseminated cancer cell dormancy: an awakening field. Nat. Rev. Cancer.

[4] Chambers AF, Groom AC, MacDonald IC (2002). Dissemination and growth of cancer cells in metastatic sites. Nat. Rev. Cancer.

[5] Allan AL, Vantyghem SA, Tuck AB, Chambers AF (2006). Tumor dormancy and cancer stem cells: implications for the biology and treatment of breast cancer metastasis. Breast Dis..

[6] Aguirre-Ghiso JA (2007). Models, mechanisms and clinical evidence for cancer dormancy. Nat. Rev. Cancer.

[7] Carcereri de Prati A (2017). Metastatic breast cancer cells enter into dormant state and express cancer stem cells phenotype under chronic hypoxia. J. Cell Biochem..

[8] Al-Hajj M, Wicha MS, Benito-Hernandez A, Morrison SJ, Clarke MF (2003). Prospective identification of tumorigenic breast cancer cells. Proc. Natl. Acad. Sci. USA.

[9] Ponti D (2005). Isolation and in vitro propagation of tumorigenic breast cancer cells with stem/progenitor cell properties. Cancer Res.

[10] Wendt MK, Taylor MA, Schiemann BJ, Schiemann WP (2011). Down-regulation of epithelial cadherin is required to initiate metastatic outgrowth of breast cancer. Mol. Biol. Cell.

[11] Gooding AJ (2017). The lncRNA BORG drives breast cancer metastasis and disease recurrence. Sci. Rep..

[12] Klein CA (2011). Framework models of tumor dormancy from patient-derived observations. Curr. Opin. Genet. Dev..

[13] Mathew R (2009). Autophagy suppresses tumorigenesis through elimination of p62. Cell.

[14] Mowers EE, Sharifi MN, Macleod KF (2017). Autophagy in cancer metastasis. Oncogene.

[15] Yang Z, Fujii H, Mohan SV, Goronzy JJ, Weyand CM (2013). Phosphofructokinase deficiency impairs ATP generation, autophagy, and redox balance in rheumatoid arthritis T cells. J. Exp. Med..

[16] Klarer AC (2014). Inhibition of 6-phosphofructo-2-kinase (PFKFB3) induces autophagy as a survival mechanism. Cancer Metab..

[17] Yan S (2017). 6-Phosphofructo-2-kinase/fructose-2,6-bisphosphatase isoform 3 spatially mediates autophagy through the AMPK signaling pathway. Oncotarget.

[18] Okar DA (2001). PFK-2/FBPase-2: maker and breaker of the essential biofactor fructose-2,6-bisphosphate. Trends Biochem. Sci..

[19] De Bock K (2013). Role of PFKFB3-driven glycolysis in vessel sprouting. Cell.

[20] Clem B (2008). Small-molecule inhibition of 6-phosphofructo-2-kinase activity suppresses glycolytic flux and tumor growth. Mol. Cancer Ther..

[21] Yalcin A (2014). 6-Phosphofructo-2-kinase (PFKFB3) promotes cell cycle progression and suppresses apoptosis via Cdk1-mediated phosphorylation of p27. Cell Death Dis..

[22] Cordero-Espinoza L, Hagen T (2013). Increased concentrations of fructose 2,6-bisphosphate contribute to the Warburg effect in phosphatase and tensin homolog (PTEN)-deficient cells. J. Biol. Chem..

[23] Atsumi T (2002). High expression of inducible 6-phosphofructo-2-kinase/fructose-2,6-bisphosphatase (iPFK-2; PFKFB3) in human cancers. Cancer Res.

[24] Morris VL, Tuck AB, Wilson SM, Percy D, Chambers AF (1993). Tumor progression and metastasis in murine D2 hyperplastic alveolar nodule mammary tumor cell lines. Clin. Exp. Metastasis.

[25] Morris VLKS (1994). Mammary carcinoma cell lines of high and low metastatic potential differ not in extravasation but in subsequent migration and growth. Clin. Exp. Metastasis.

[26] Rak JW, McEachern D, Miller FR (1992). Sequential alteration of peanut agglutinin binding-glycoprotein expression during progression of murine mammary neoplasia. Br. J. Cancer.

[27] Naumov GN (2002). Persistence of solitary mammary carcinoma cells in a secondary site: a possible contributor to dormancy. Cancer Res..

[28] Shibue T, Weinberg RA (2009). Integrin beta1-focal adhesion kinase signaling directs the proliferation of metastatic cancer cells disseminated in the lungs. Proc. Natl. Acad. Sci. USA.

[29] Vera-Ramirez L, Vodnala SK, Nini R, Hunter KW, Green JE (2018). Autophagy promotes the survival of dormant breast cancer cells and metastatic tumour recurrence. Nat. Commun..

[30] Wendt MK, Allington TM, Schiemann WP (2009). Mechanisms of the epithelial-mesenchymal transition by TGF-beta. Future Oncol..

[31] Pulaski, B. A. & Ostrand-Rosenberg, S. Mouse 4T1 breast tumor model. *Curr. Protoc. Immunol.* **39**, 20.2.1–20.2.16 (2001). 10.1002/0471142735.im2002s39 18432775

[32] Wendt MK, Smith JA, Schiemann WP (2010). Transforming growth factor-beta-induced epithelial-mesenchymal transition facilitates epidermal growth factor-dependent breast cancer progression. Oncogene.

[33] Zhang X (2013). A renewable tissue resource of phenotypically stable, biologically and ethnically diverse, patient-derived human breast cancer xenograft models. Cancer Res.

[34] Smid M (2008). Subtypes of breast cancer show preferential site of relapse. Cancer Res.

[35] Ali HR (2011). Cancer stem cell markers in breast cancer: pathological, clinical and prognostic significance. Breast Cancer Res.

[36] Visvader JE, Lindeman GJ (2006). Mammary stem cells and mammopoiesis. Cancer Res.

[37] Correa RJ, Peart T, Valdes YR, DiMattia GE, Shepherd TG (2012). Modulation of AKT activity is associated with reversible dormancy in ascites-derived epithelial ovarian cancer spheroids. Carcinogenesis.

[38] Gump JM, Thorburn A (2014). Sorting cells for basal and induced autophagic flux by quantitative ratiometric flow cytometry. Autophagy.

[39] Sizemore ST (2014). Hypomethylation of the MMP7 promoter and increased expression of MMP7 distinguishes the basal-like breast cancer subtype from other triple-negative tumors. Breast Cancer Res. Treat..

[40] Kimura T, Takabatake Y, Takahashi A, Isaka Y (2013). Chloroquine in cancer therapy: a double-edged sword of autophagy. Cancer Res.

[41] Maycotte P (2012). Chloroquine sensitizes breast cancer cells to chemotherapy independent of autophagy. Autophagy.

[42] Lippai M, Low P (2014). The role of the selective adaptor p62 and ubiquitin-like proteins in autophagy. Biomed. Res. Int..

[43] Bjorkoy G (2005). p62/SQSTM1 forms protein aggregates degraded by autophagy and has a protective effect on Huntingtin-induced cell death. J. Cell Biol..

[44] Seibenhener ML (2004). Sequestosome 1/p62 is a polyubiquitin chain binding protein involved in ubiquitin proteasome degradation. Mol. Cell Biol..

[45] Lu Z (2008). The tumor suppressor gene ARHI regulates autophagy and tumor dormancy in human ovarian cancer cells. J. Clin. Invest.

[46] Whelan KA (2017). Autophagy supports generation of cells with high CD44 expression via modulation of oxidative stress and Parkin-mediated mitochondrial clearance. Oncogene.

[47] McGowan PM, Kirstein JM, Chambers AF (2009). Micrometastatic disease and metastatic outgrowth: clinical issues and experimental approaches. Future Oncol..

[48] Hue L, Rider MH (1987). Role of fructose 2,6-bisphosphate in the control of glycolysis in mammalian tissues. Biochem. J..

[49] Domenech E (2015). AMPK and PFKFB3 mediate glycolysis and survival in response to mitophagy during mitotic arrest. Nat. Cell Biol..

[50] Thorburn A, Thamm DH, Gustafson DL (2014). Autophagy and cancer therapy. Mol. Pharm..

[51] Yalcin A (2017). 6-Phosphofructo-2-kinase/fructose 2,6-bisphosphatase-3 is required for transforming growth factor beta1-enhanced invasion of Panc1 cells in vitro. Biochem. Biophys. Res. Commun..

[52] Bryson, B. L., Junk, D. J., Cipriano, R. & Jackson, M. W. STAT3-mediated SMAD3 activation underlies Oncostatin M-induced senescence. *Cell Cycle* **16**, 319–334 (2016). 10.1080/15384101.2016.1259037 PMC5324753 27892764

[53] Johannessen CM (2010). COT drives resistance to RAF inhibition through MAP kinase pathway reactivation. Nature.

[54] Hu Y, Smyth GK (2009). ELDA: extreme limiting dilution analysis for comparing depleted and enriched populations in stem cell and other assays. J. Immunol. Methods.

[55] Taylor MA, Amin JD, Kirschmann DA, Schiemann WP (2011). Lysyl oxidase contributes to mechanotransduction-mediated regulation of transforming growth factor-beta signaling in breast cancer cells. Neoplasia.

