# Supplementary material for: Autophagy inhibition elicits emergence from metastatic dormancy by inducing and stabilizing Pfkfb3 expression
Source: Nat Commun. 2019 Aug 14;10:3668. doi: 10.1038/s41467-019-11640-9 (PMC6694140; doi:10.1038/s41467-019-11640-9)

Supplementary Information

Autophagy inhibition elicits emergence from metastatic dormancy by inducing and stabilizing Pfkfb3 expression

Flynn *et al.*

Supplementary Table 1. PDX Models. The model number, breast cancer subtype, rate of xenograft metastasis, site of metastasis in the patient, and nodal status of the patient for each PDX model is described.

Model	Breast Cancer Subtype	Rate of xenograft metastasis	Patient Metastatic Site	Nodal Status of Patient
BCM-3613	HER2+/ER-/PR-	23.8%	Brain	Not reported
BCM-4664	TNBC	0%	None	+
BCM-3963	HER2+/ER-/PR-	13.6%	Brain	+
BCM-4013	TNBC	21.4%	None	+
BCM-2664	TNBC	7.1%	None	-
BCM-4913	TNBC	0%	None	+
BCM-3887	TNBC	14.3%	Brain	-
BMC-3204	TNBC	28.6%	None	-

Supplementary Table 2. shRNAs. The gene, catalog number from Sigma Aldrich, sequence, and the identifying shRNA number are described for each shRNA used in this study.

Gene	Catalog Number	Sequence	shRNA designation
Atg3	Sigma Aldrich Cat. # TRCN0000247441	5'-CCGGTGTGACCATTGACCAT ATTTACTCGAGTAAATATGGTCAAT GGTCACATTTTTG	shAtg3
Atg7	Sigma Aldrich Cat. #TRCN0000375444	5'-CCGGGCCAACATCCCTGGAT ACAAGCTCGAGCTTGTATCCAGGG ATGTTGGCTTTTTG	shAtg7_sh1
	Sigma Aldrich Cat. # TRCN0000305993	5'-CCGGGCAGTGATGACCGCA TGAATGCTCGAGCATTCATGCGGT CATCACTGCTTTTTG	shAtg7_sh2
	Sigma Aldrich Cat. # TRCN0000305991	5'-CCGGTTCTGTCACGGTTCGA TAATGCTCGAGCATTATCGAACCG TGACAGAATTTTTG	shAtg7_sh3
p62/ SQSTM1	Sigma Aldrich Cat. # TRCN0000238134	5'-CCGGCACCCTCCACCATTGT GATAGCTCGAGCTATCACAATGGT GGAGGGTGTTTTTG	shp62_sh1
	Sigma Aldrich Cat. # TRCN0000238135	5'-CCGGTGTGGTGGGAACTCG CTATAACTCGAGTTATAGCGAGTT CCCACCACATTTTTG	shp62_sh2
FIP200	Sigma Aldrich Cat. # TRCN0000331566	5'-CCGGCCAACTTTAACACAGT CTTAACTCGAGTTAAGACTGTGTTA AAGTTGGTTTTTG	shFIP200_sh1
	Sigma Aldrich Cat. # TRCN0000304383	5'-CCGGGATAATGCATACTCAA CATTGCTCGAGCAATGTTGAGTAT GCATTATCTTTTTG	shFIP200_sh2
	Sigma Aldrich Cat. # TRCN0000304384	5'-CCGGATTGCATGCAGATAAT CATAACTCGAGTTATGATTATCTGC ATGCAATTTTTTG	shFIP200_sh3
Pfkfb3	Sigma Aldrich Cat. # TRCN0000025418	5'-CCGGGTGTTCAATGTGGGA GAGTATCTCGAGATACTCTCCCAC ATTGAACACTTTTT	shPfkfb3

Supplementary Table 3. Cell Line Culture Conditions for each human and murine cell line are described.

Cell Line	Base Media	Supplements
D2.HAN (D2.OR and D2.A1)	DMEM	10% FBS and 1% Pen/Strep
4T1 Series (67NR, 4T07, 4T1)	DMEM	10% FBS and 1% Pen/Strep
MDA-MB-231	DMEM	10% FBS and 1% Pen/Strep
BT474	DMEM	10% FBS and 1% Pen/Strep
MCF7	DMEM	10% FBS, 1 μg/mL insulin (Sigma Aldrich) and 1% Pen/Strep
T47D	RPMI-1640	10% FBS, 1 μg/mL insulin (Sigma Aldrich), and 1% Pen/Strep
BT549	RPMI-1640	10% FBS, 1 μg/mL insulin (Sigma Aldrich), and 1% Pen/Strep
HCC1806	RPMI-1640	10% FBS and 1% Pen/Strep

Supplementary Table 4. RT-PCR Primers. Gene, species, and primer sequences for each primer used in this study.

Gene Name	Species	Forward Primer	Reverse Primer
Pfkfb3	Mouse	5'-CGGGAGAGGTCAGAG AACAACATGA	5'-TTGGCCTCGAGAAGATGA GC
PFKFB3	Human	5'-GATGCCCTTCAGGAAA GCCT	5'-TCCCCGACGTTGAACACT TT
p62/ SQSTM1	Human	5'-CTATGGCGTCGCTCAC CGT	5'-CCGTCCTCATCGCGGTAG TG
Gapdh	Mouse	5'-CAACTTTGGCATTGTG GAAGGGCTC	5'-GCAGGGATGATGTTCTGG GC AGC
GAPDH	Human	5'-TCCATGACAACTTTGGT ATCGT	5'-AGTAGAGGCAGGGATGA TGTT

Supplementary Table 5. Antibodies**.** The antibody, catalog number and staining condition dilution as well as the application for each antibody used in this study.

**Antibody**	**Application**	**Dilution**	**Catalog Number**
Pfkfb3	Western Blotting	1:1000	Abcam (ab181861)
p62/ SQSTM1	Western Blotting	1:1000	Abcam (ab56416)
LC3B	Western Blotting	1:2500	Novus Bio (NB100-2220)
Atg3	Western Blotting	1:1000	Cell Signaling (# 3415)
Atg5	Western Blotting	1:1000	Cell Signaling (#12994)
Atg12	Western Blotting	1:1000	Cell Signaling (#4180)
Beclin-1	Western Blotting	1:1000	Cell Signaling (#3495)
GFP	Western Blotting	1:1000	Abcam (ab6556)
FLAG	Western Blotting/ Co-immunoprecipitation	1:750	Sigma (#F3165)
HA	Western Blotting/ Co-immunoprecipitation	1:750	Abcam (ab16918)
β-Actin	Western Blotting	1:20,000	Sigma
Pfkfb3	IHC	1:250	Abcam (ab181861)
p62/ SQSTM1	IHC	50 μg/mL	Abcam (ab91526)
LC3B	IHC	1:1000	Novus Bio (NB100-2220)
Atg3	IHC	2.5 μg/mL	Thermo Fisher (720056)
LC3B-AF647	Immunofluorescence	1:250	Abcam (ab225383)
LAMP1-Cy3	Immunofluorescence	1:150	Abcam (ab67283)
CD24-BV421	Flow cytometry	1:1000	BD (562563)
CD49f-PE	Flow cytometry	1:1000	Invitrogen (12-0495-82)

Supplementary Figure 1: Flynn et al.

a

Gene Name	Gene Symbol	Fold-change
ABL	Abl1	1.54
Aldehyde Dehydrogenase 1 Family Member A3	Aldh1a3	0.01
Calreticulin	Calr	0.48
Cyclin A2	Ccna2	1.88
Cyclin B1	Ccnb1	3.73
Cyclin E1	Ccne1	2.94
CD44	CD44	0.61
Cell Division Cycle 25C	Cdc25c	2.28
Cyclin-dependent Kinase 2	Cdk2	2.10
Cyclin-dependent Kinase 4	Cdk4	2.23
Cyclin-dependent Kinase Inhibitor 1	Cdkn1a	0.30
Checkpoint Kinase 1	Chek1	4.03
Checkpoint Kinase 2	Chek2	1.78
Cbp/p300-interacting Transactivator 2	Cited2	6.10
Collagen type 1, alpha 1	Col1a1	0.01
Collagen type 3, alpha 1	Col3a1	0.31
Cellular Repressor of E1A-stimulated Genes 1	Creg1	4.02
E2F Transcription Factor 1	E2f1	3.63
E2F Transcription Factor 3	E2f3	1.75
Early Growth Response Protein 1	Egr1	0.37
Ets2	Ets2	2.38
Fibronectin	Fn1	3.61
Growth Arrest & DNA-damage Inducible Alpha	Gadd45a	0.24
Inhibitor of DNA-binding 1	Id1	0.42
Interferon Gamma	Infg	0.09
Insulin Growth Factor 1	Igf1	0.01
Insulin Growth Factor-binding Protein 5	Igfbp5	0.01
Interferon Regulatory Factor 3	Irf3	1.91
Interferon Regulatory Factor 5	Irf5	15.03
Interferon Regulatory Factor 7	Irf7	6.91
Mouse Double Minute 2	Mdm2	1.99
MORC3	Morc3	1.99
Myc	Myc	9.10
NADPH Oxidase 4	Nox4	2.89
Proliferating Cell Nuclear Antigen	Pcna	3.39
Urokinase	Plau	0.20
Phosphatase & Tensin Homolog	Pten	0.38
Retinoblastoma-like Protein 1	Rbl1	2.55
Superoxide Dismutase 1	Sod1	4.82
Secreted Protein Acidic & Cysteine Rich	Sparc	0.18
T-box Transcription Factor 2	Tbx2	2.44
T-box Transcription Factor 3	Tbx3	45.67
Telomeric Repeat Binding Factor 2	Terf2	1.89
Telomerase	Tert	12.22
Tumor protein 53	Trp53	0.01
Twist BHLH Transcription Factor 1	Twist1	1.82

b

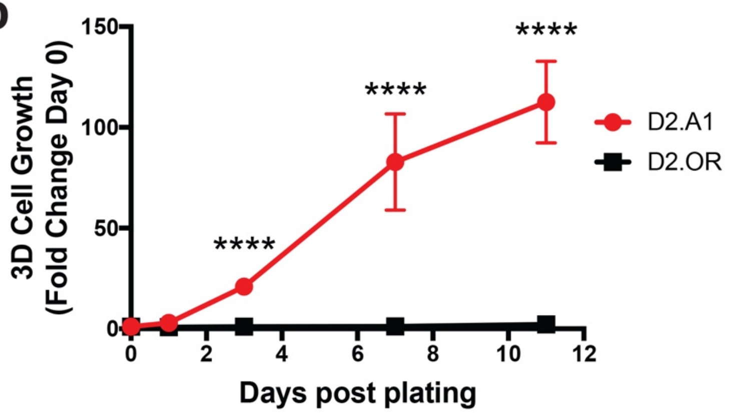

Supplementary Figure 1. D2.OR cells remain dormant in a 3D tissue culture system and express numerous senescence associated genes. **a** Senescence-associated genes are differentially expressed between dormant D2.OR and metastatic D2.A1 cells. Data are gene expression levels in D2.A1 cells normalized to D2.OR cells using a 1.5-fold cut-off. **b** D2.OR cells exhibit minimal

outgrowth in 3D-cultures as compared to their metastatic D2.A1 counterparts. Data represent mean (±STDEV) of biological replicates performed in triplicate (*****P*≤0.0001). This experiment was independently repeated for a total of 3 experiments, all with similar results.

Supplementary Figure 2: Flynn et al.

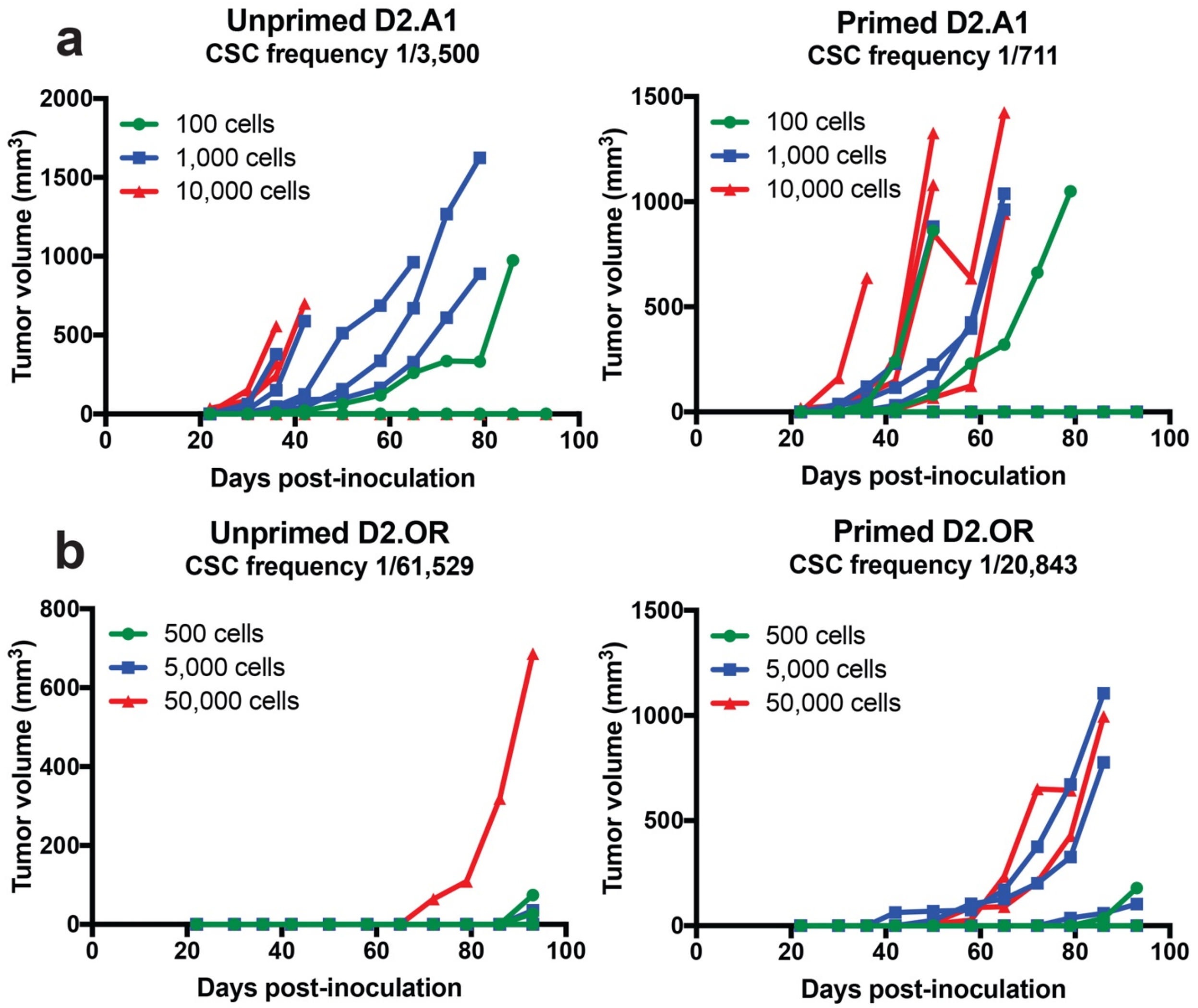

Supplementary Figure 2. Priming increases the stem cell frequency and decreases the latency of mammary fat pad tumor formation *in vivo*. D2.OR and D2.A1 primed and unprimed cells were inoculated into the mammary fat pads of BALB/C mice (Fig. 1). **a** Metastatic D2.A1 cells exhibit early outgrowth from the mammary fat pad. **b** Priming of dormant D2.OR cells decreases tumor latency while increasing stem cell frequency as determined by ELDA: Extreme Limiting Dilution Analysis http://bioinf.wehi.edu.au/software/elda/. The ELDA software uses a test to evaluate differences between population groups (analogous to one-way ANOVA) in Fig. 1C. Data represent n=5 mice/group.

Supplementary Figure 3: Flynn et al

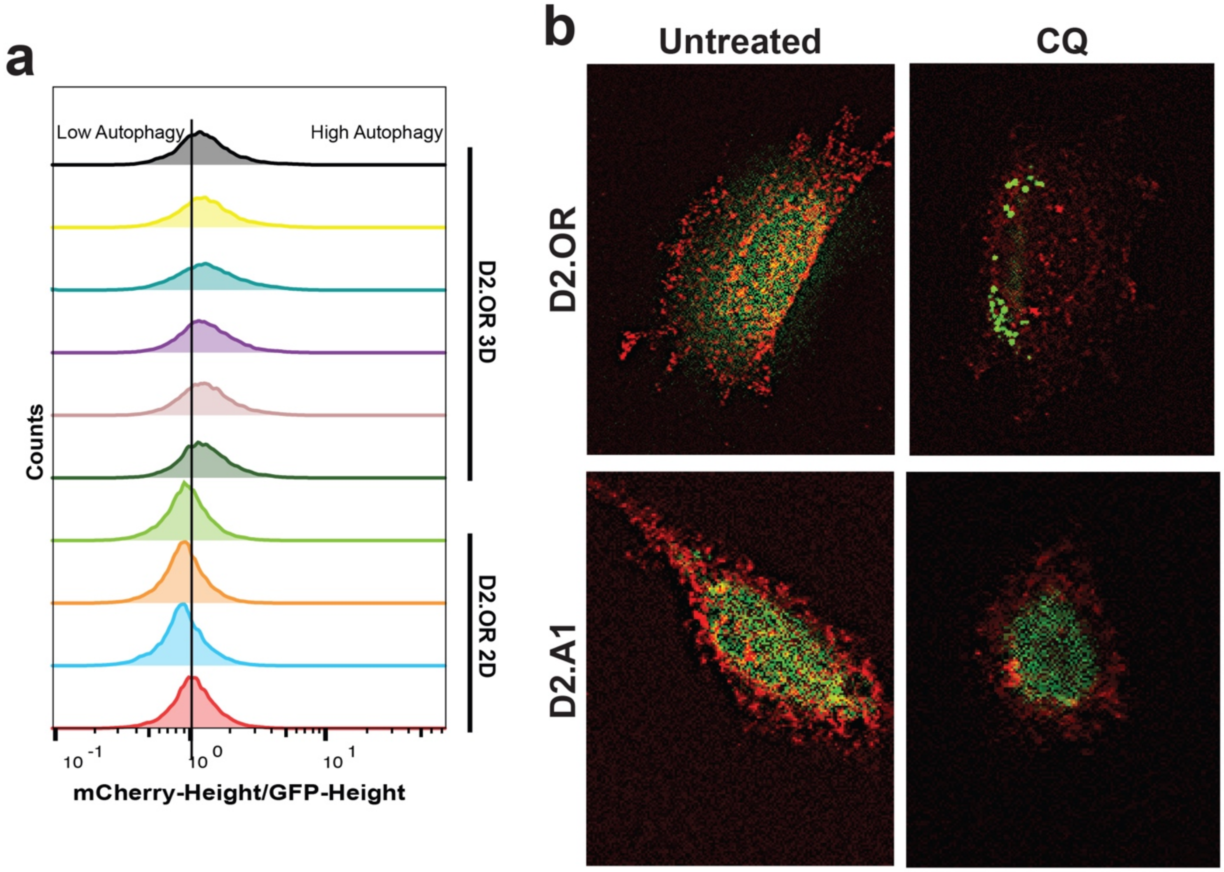

Supplementary Figure 3. Measuring autophagic flux in dormant cells. **a** Autophagy flux mCherry-Height:GFP-Height ratio in “primed” D2.OR (3D) and “unprimed” D2.OR (2D) cells. **b** Immunofluorescence of LAMP1 (green) and LC3B (red) colocalization in dormant D2.OR and metastatic D2.A1 cells untreated and treated with chloroquine (50 μM; CQ).

Supplementary Figure 4: Flynn et al

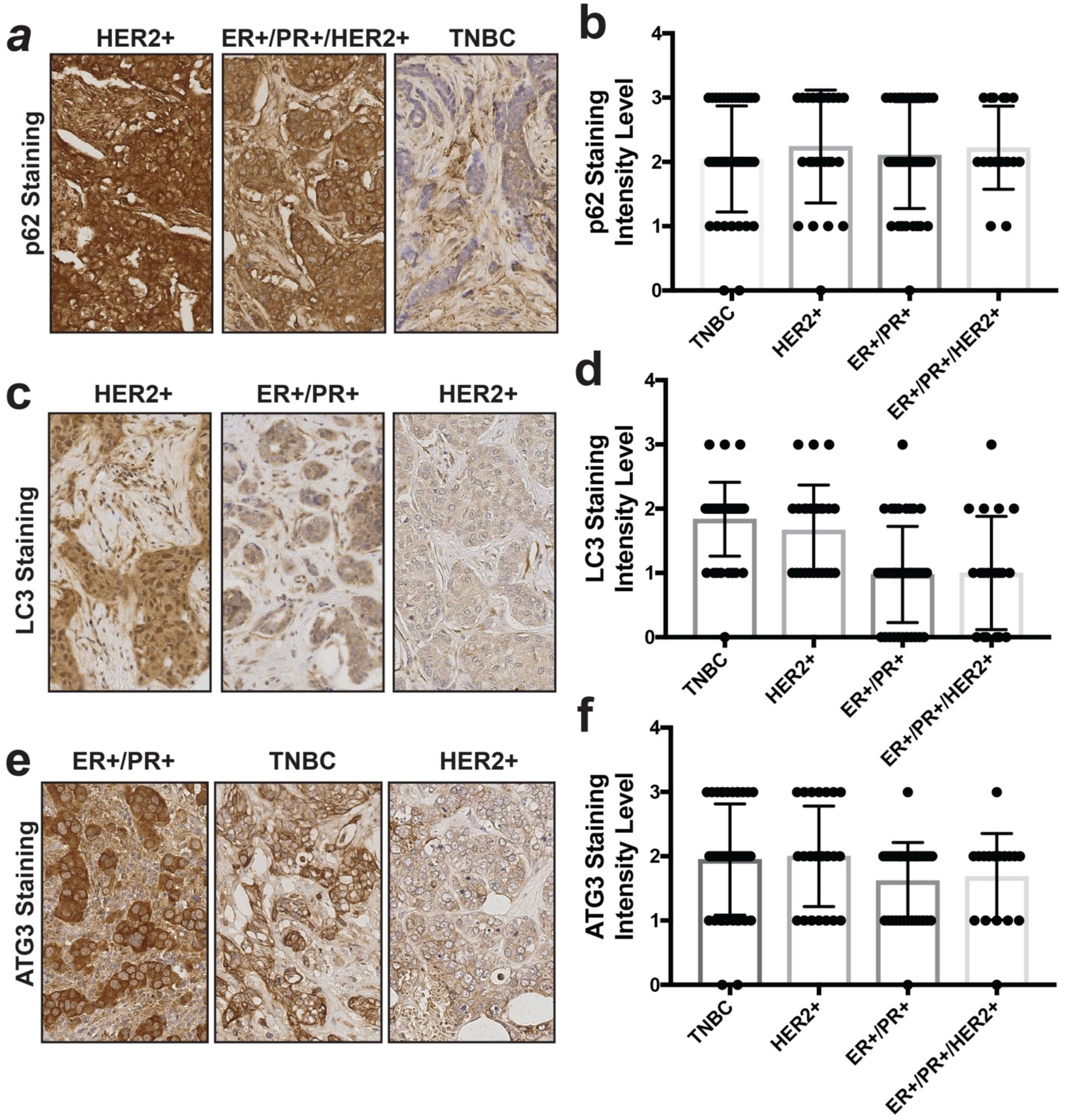

Supplementary Figure 4. Assessing autophagy status in human breast cancer tumor samples. The Case Comprehensive Cancer Center Breast Cancer TMA was stained for p62/SQSTM1, LC3 and ATG3. Representative IHC staining for these antigens are shown in panels **a, c,** and **e** Quantification of staining intensity levels of each of the cores on the TMA are highlighted in panels **b, d** and **f** 100 breast cancer sample cores were evaluated for each antigen.

Supplementary Figure 5: Flynn et al

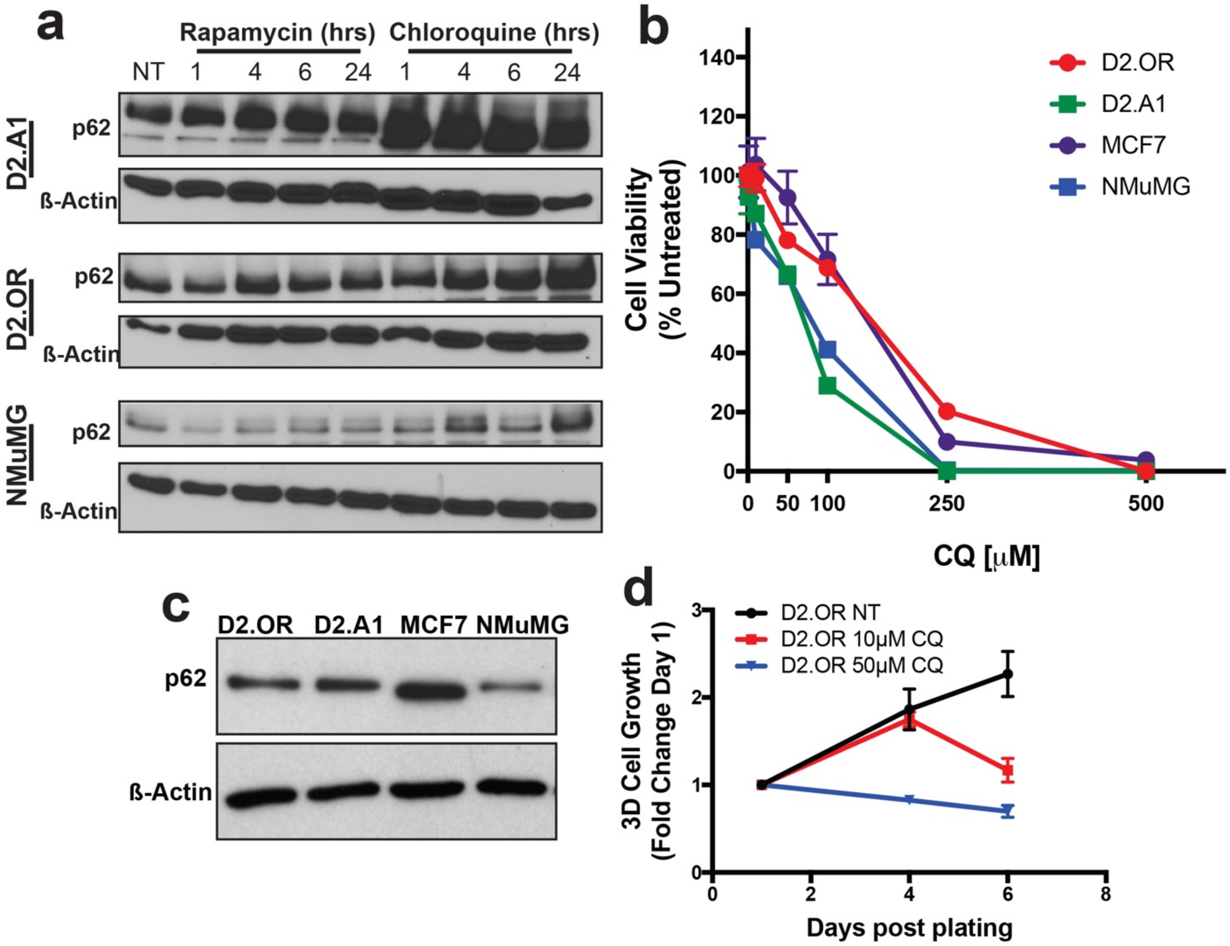

Supplementary Figure 5. Modulating autophagy in dormant cells. Genetic inhibition of autophagy in dormant cells. **a** NMuMG, D2.OR, and D2.A1 cells were incubated in the absence (NT) or presence of either rapamycin (50 nM) or chloroquine (CQ) (10 μM) for 0-24 hours (hrs) as indicated. Alterations in p62/ SQSTM1 expression was monitored by immunoblotting. **b** D2.OR, D2.A1, MCF7, and NMuMG cells were incubated in the absence or presence of the autophagy inhibitor, chloroquine (CQ) under serum reduced (1%) conditions as indicated. Differences in cell viability were quantified by CellTiter-Glo Assay. Data are the mean (± S.E.M; n=3). **c** NMuMG cells are highly autophagic, while MCF7 cells exhibit low levels of basal autophagy. **d** Growth of dormant D2.OR cells in the absence or presence of \ 10 μM or 50 μM CQ in 3D-culture systems.

Supplementary Figure 6: Flynn et al

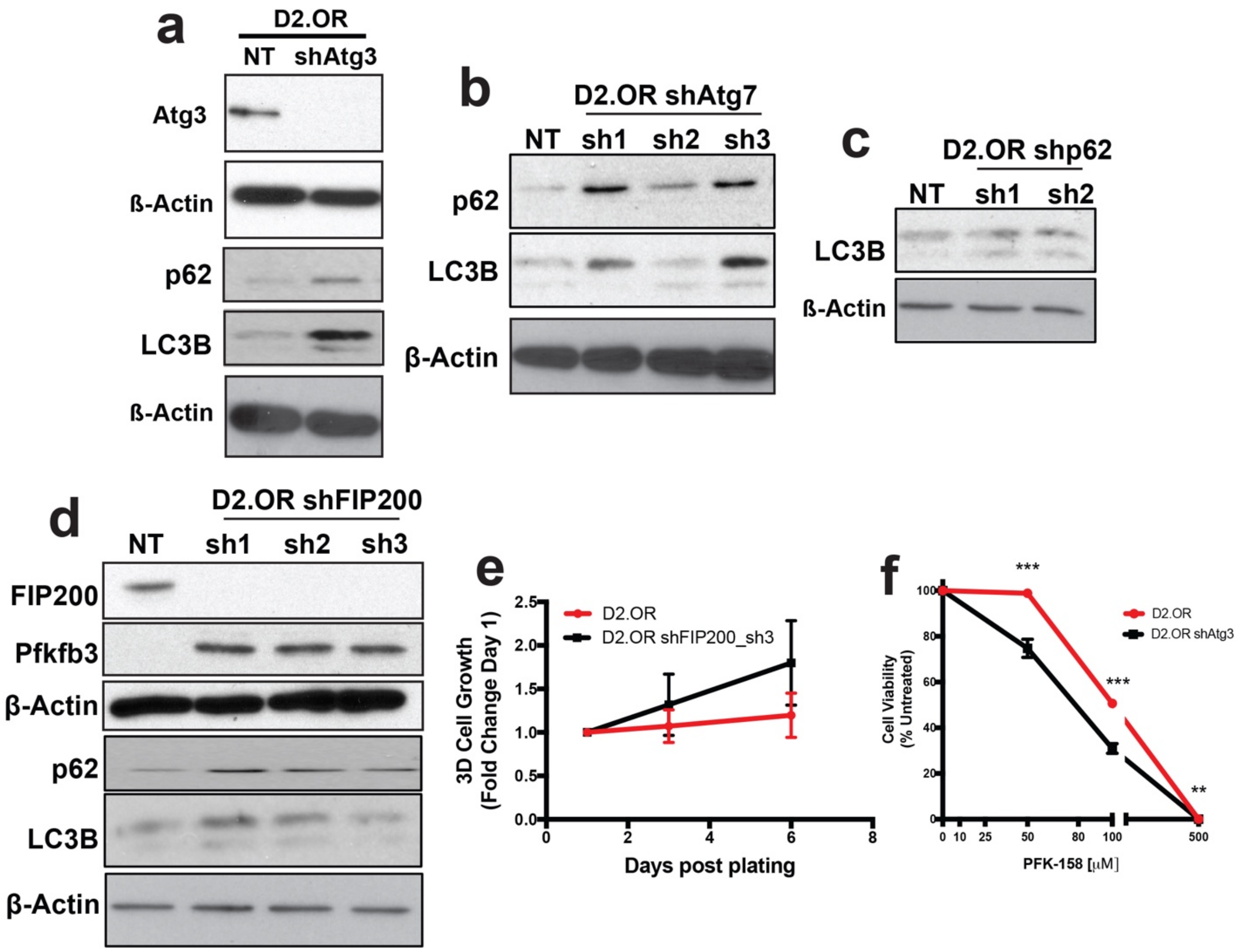

Supplementary Figure 6: Autophagy inhibition in dormant D2.OR cells. Depleting the expression of Atg3 (**a**; shAtg3), Atg7 (**b**; shAtg7), p62/ SQSTM1 (**c**; shp62), and FIP200 (**d**; shFIP200) inhibits autophagy as determined by immunoblotting for p62/SQSTM1 and LC3B. **d** FIP200-deficiency promotes aberrant Pfkfb3 expression in D2.OR cells and **e,** induces their outgrowth in 3D-cultures. Data represent mean (±STDEV of biological replicates performed in triplicate. This experiment was independently repeated for a total of three experiments, all with similar results. **(e)**. **f** Depleting Atg3 expression renders D2.OR cells more sensitive to the Pfkfb3 inhibitor, PFK158. Data represent mean (±STDEV) of biological replicates performed in triplicate (** *P*≤ 0.01 and ****P*≤0.001). This experiment was independently repeated for a total of 3 experiments, all with similar results.

Supplementary Figure 7: Flynn et al

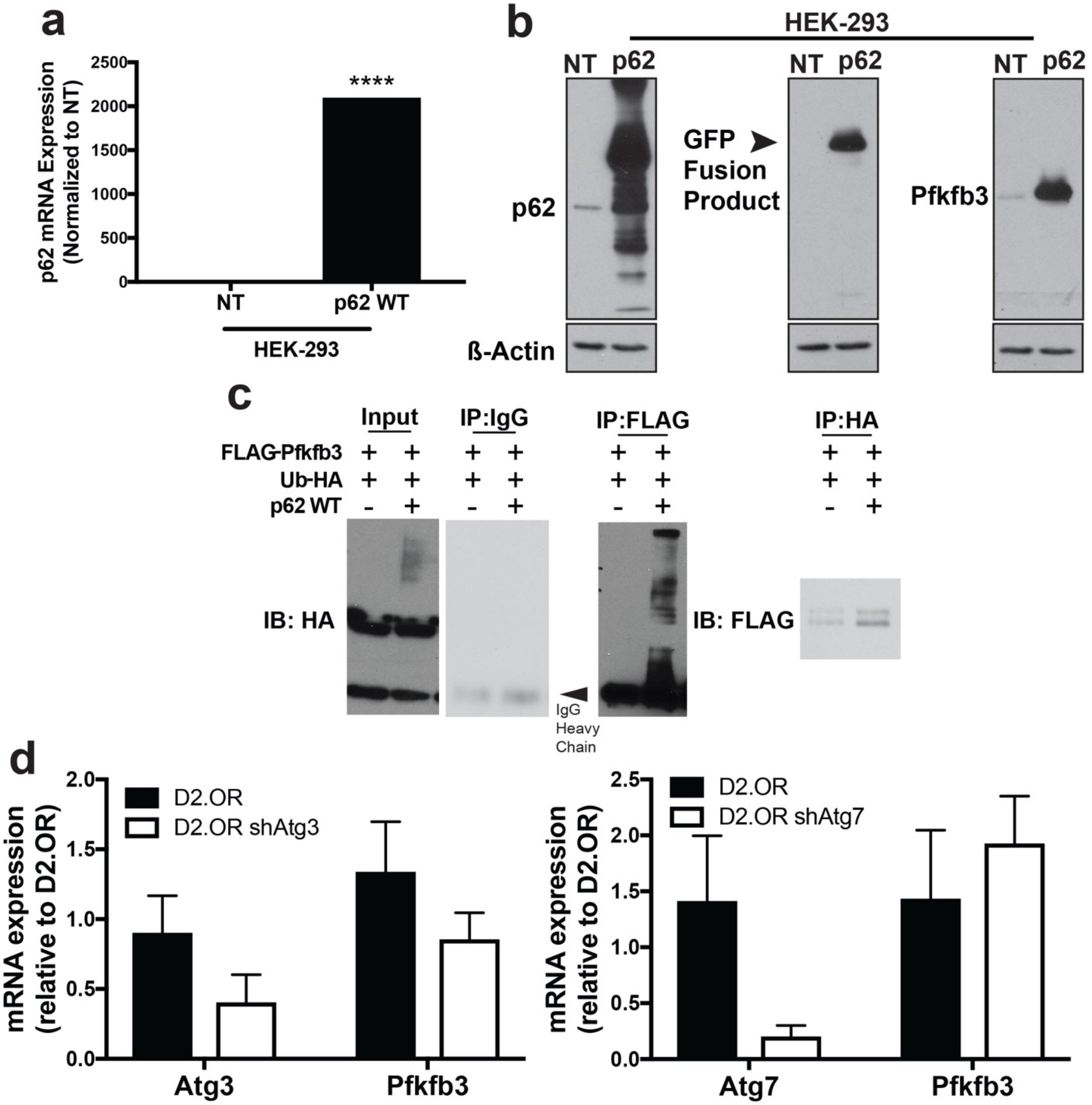

Supplementary Figure 7. p62/SQSTM1 binds to ubiquitinated Pfkfb3. HEK-293 cells were transiently transfected with empty vector or p62/ SQSTM1 fused to GFP (pDestEGFP-p62). **a** p62 mRNA expression. Data represent mean (±STDEV) of replicates performed in triplicate (**** *P*<0.0001). This experiment was independently repeated for a total of 3 experiments, all with similar results. **b** Alterations in p62/SQSTM1, GFP, and Pfkfb3 expression were monitored by immunoblotting. **c** Immunoblot (IB) analysis of HEK-293 cells transiently transfected with FLAG-tagged Pfkfb3, HA-tagged Ubiquitin (Ub-HA), or GFP-tagged p62/SQSTM1 mutants. Subsequently, anti-FLAG immunocomplexes were captured and immunoblotted with antibodies against FLAG or HA as indicated. **d** Knockdown of Atg3 and Atg7 does not significantly impact the expression levels of Pfkfb3 mRNA after 8 passages following autophagy inactivation.

Supplementary Figure 8: Flynn et al

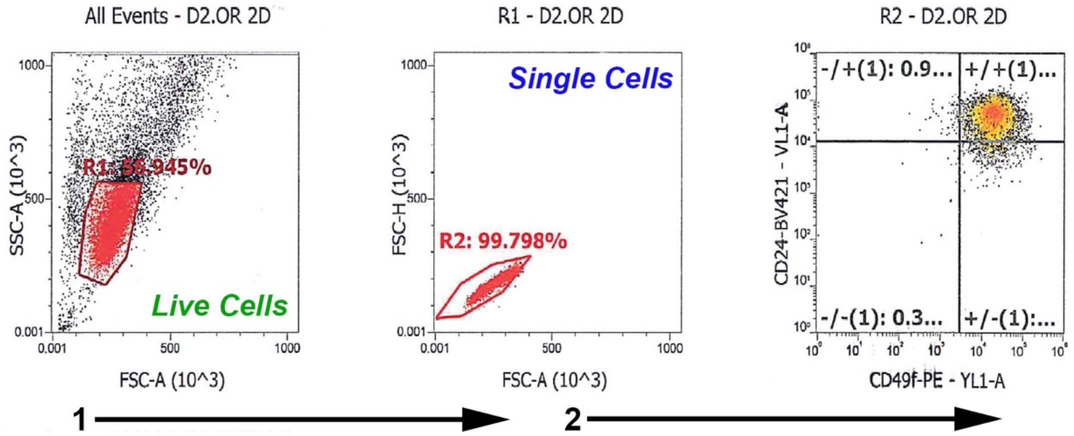

Supplementary Figure 8. Gating strategy.

Supplementary Figure 9. Uncropped gel images

Figure 3a

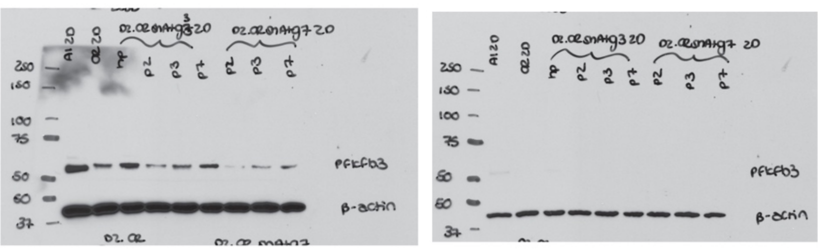

Figure 3f

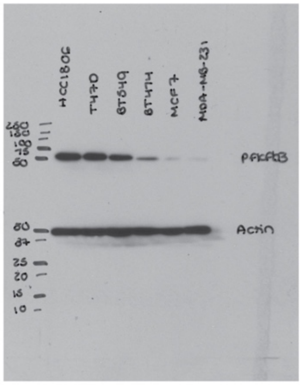

Figure 4b

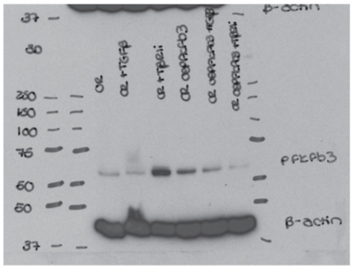

Figure 4c

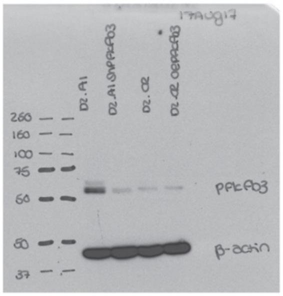

Figure 5b

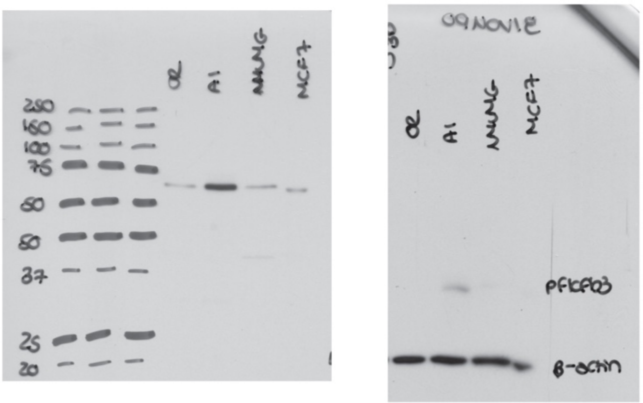

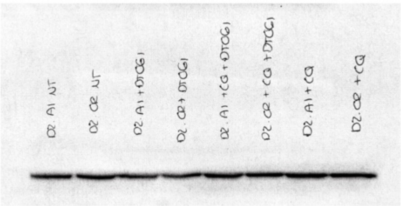

Actin

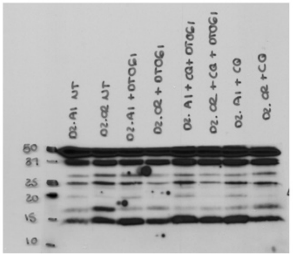

LC3B

Figure 6a

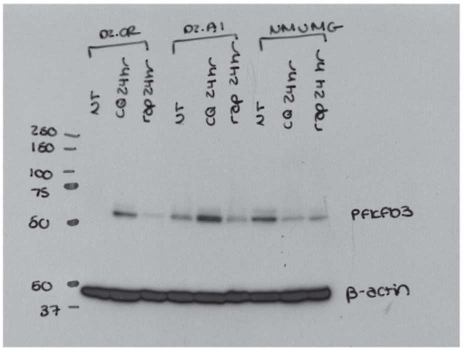

Figure 6b

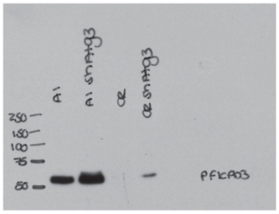

Figure 6b

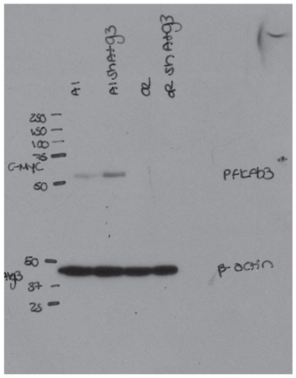

Figure 6c

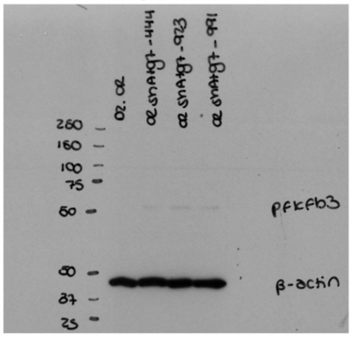

Figure 6c

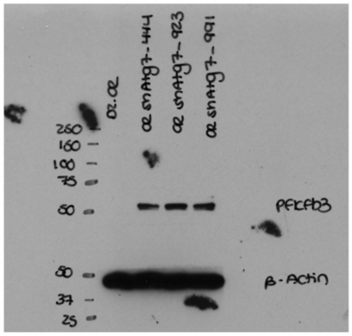

Figure 6c

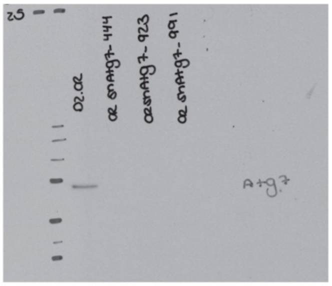

Figure 6d

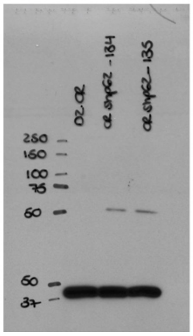

Figure 6d

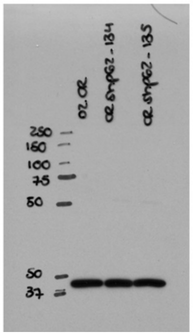

Figure 6d

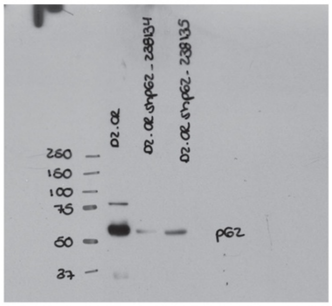

Figure 7c

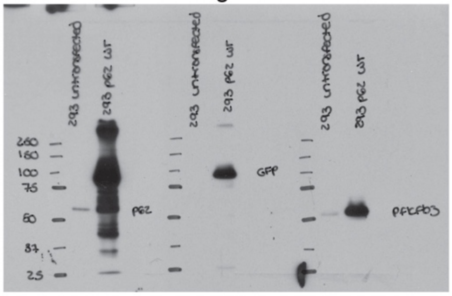

Figure 7c

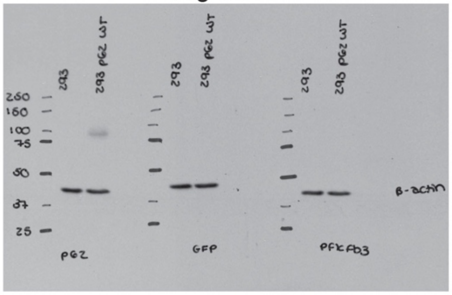

Figure 7d

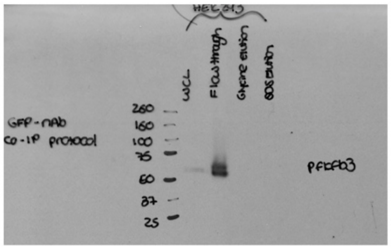

Figure 7d

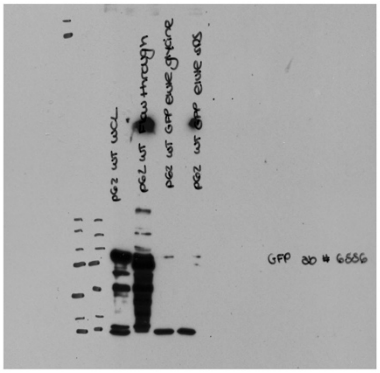

Figure 7d

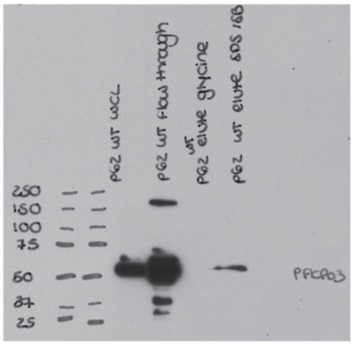

Figure 7e

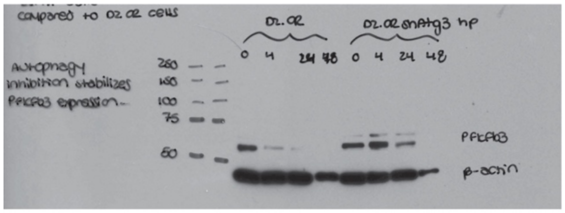

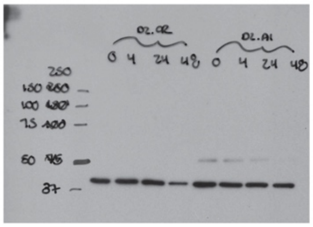

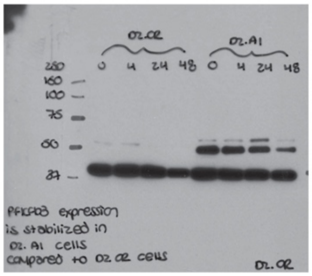

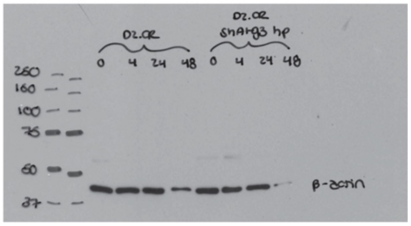

Figure 7f

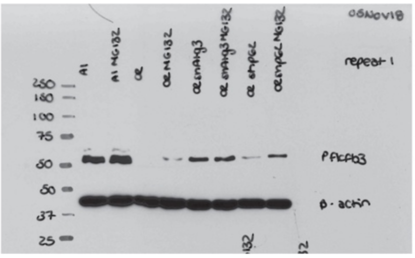

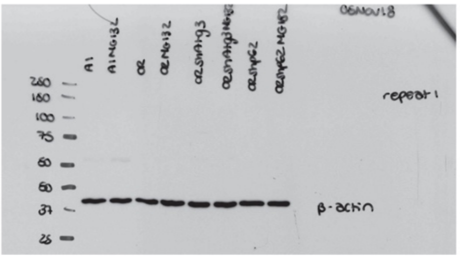

Supplementary Figure 6a

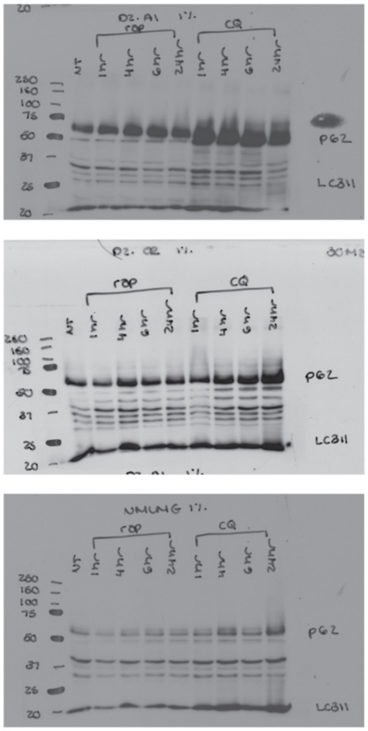

Supplementary Figure 6a

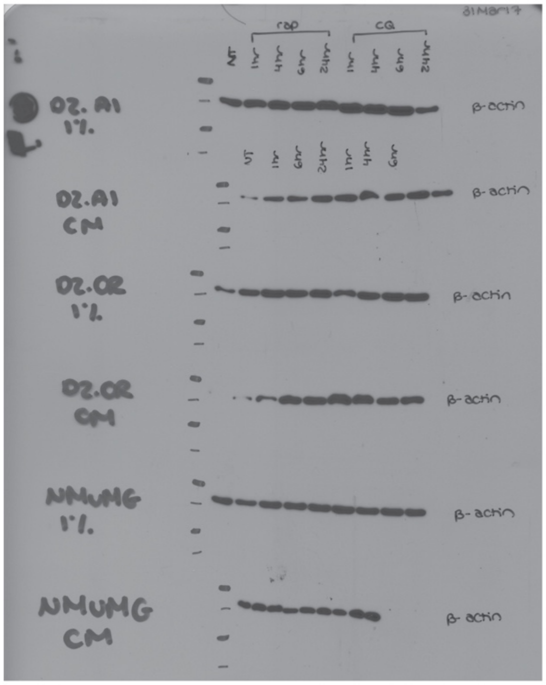

Supplementary Figure 6c

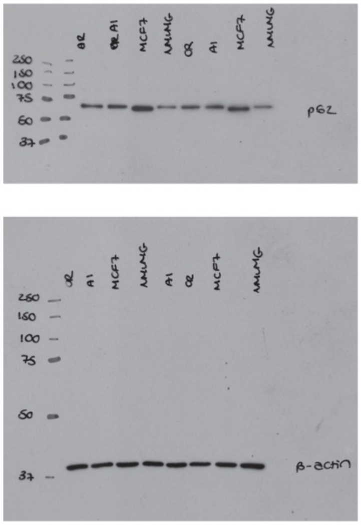

Supplementary Figure 6d

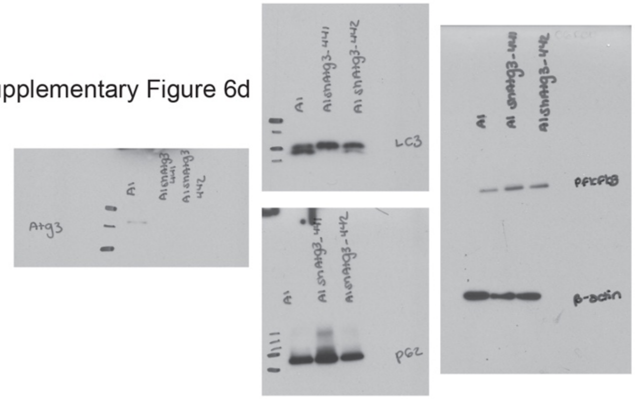

Supplementary Figure 6e

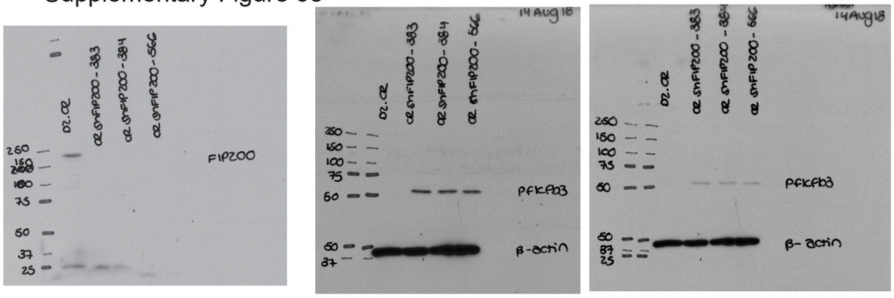

Supplement: Supplementary file 1 — Supplementary Information [file 41467_2019_11640_MOESM1_ESM.pdf]
