# Supplementary material for: Autophagy inhibition elicits emergence from metastatic dormancy by inducing and stabilizing Pfkfb3 expression
Source: Nat Commun. 2019 Aug 14;10:3668. doi: 10.1038/s41467-019-11640-9 (PMC6694140; doi:10.1038/s41467-019-11640-9)
Supplement: Supplementary file 2 — Reporting Summary [file 41467_2019_11640_MOESM2_ESM.pdf]

nature research

Corresponding author(s): William P. Schiemann

Last updated by author(s): Jul 16, 2019

Reporting Summary

Nature Research wishes to improve the reproducibility of the work that we publish. This form provides structure for consistency and transparency in reporting. For further information on Nature Research policies, see Authors & Referees and the Editorial Policy Checklist.

Statistics

For all statistical analyses, confirm that the following items are present in the figure legend, table legend, main text, or Methods section.

n/a	Confirmed	
☐	☒	The exact sample size (*n*) for each experimental group/condition, given as a discrete number and unit of measurement
☐	☒	A statement on whether measurements were taken from distinct samples or whether the same sample was measured repeatedly
☐	☒	The statistical test(s) used AND whether they are one- or two-sided<br>*Only common tests should be described solely by name; describe more complex techniques in the Methods section.*
☒	☐	A description of all covariates tested
☐	☒	A description of any assumptions or corrections, such as tests of normality and adjustment for multiple comparisons
☐	☒	A full description of the statistical parameters including central tendency (e.g. means) or other basic estimates (e.g. regression coefficient) AND variation (e.g. standard deviation) or associated estimates of uncertainty (e.g. confidence intervals)
☐	☒	For null hypothesis testing, the test statistic (e.g. *F*, *t*, *r*) with confidence intervals, effect sizes, degrees of freedom and *P* value noted<br>*Give P values as exact values whenever suitable.*
☒	☐	For Bayesian analysis, information on the choice of priors and Markov chain Monte Carlo settings
☒	☐	For hierarchical and complex designs, identification of the appropriate level for tests and full reporting of outcomes
☒	☐	Estimates of effect sizes (e.g. Cohen's *d*, Pearson's *r*), indicating how they were calculated

Our web collection on statistics for biologists contains articles on many of the points above.

Software and code

Policy information about availability of computer code

Data collection	Keyence, Attune, GloxMax, Ivis Spectrum
Data analysis	Image J (NIH; Version:2.0.0-rc-43/1.50e), Prism 7 (Version 7.0d), Ingenuity Pathway Analysis, Microsoft Excel, Attune NxT Version 3.1

For manuscripts utilizing custom algorithms or software that are central to the research but not yet described in published literature, software must be made available to editors/reviewers. We strongly encourage code deposition in a community repository (e.g. GitHub). See the Nature Research guidelines for submitting code & software for further information.

Data

Policy information about availability of data

All manuscripts must include a data availability statement. This statement should provide the following information, where applicable:

- Accession codes, unique identifiers, or web links for publicly available datasets
- A list of figures that have associated raw data
- A description of any restrictions on data availability

We have provided this information in the Data Availability statement

Field-specific reporting

Please select the one below that is the best fit for your research. If you are not sure, read the appropriate sections before making your selection.

☒ Life sciences ☐ Behavioural & social sciences ☐ Ecological, evolutionary & environmental sciences

For a reference copy of the document with all sections, see nature.com/documents/nr-reporting-summary-flat.pdf

Life sciences study design

All studies must disclose on these points even when the disclosure is negative.

Sample size	All quantitative experiments were repeated three times unless otherwise noted. Student t-tests were used for pair-wise comparison.
Data exclusions	Data points were not excluded from analysis
Replication	Independent replicates were repeated as noted.
Randomization	For breast cancer xenograft studies, the mice were randomized into (i) 4 groups (n=3 per group) for priming analyses; (ii) 12 groups (n=5 per group) for limiting-dilution assays (LDA); and (iii) 2 groups (n=10 per group) for Atg3-deficient cells.
Blinding	Analysis of immunohistochemistry experiments were performed in a blinded fashion.

Reporting for specific materials, systems and methods

We require information from authors about some types of materials, experimental systems and methods used in many studies. Here, indicate whether each material, system or method listed is relevant to your study. If you are not sure if a list item applies to your research, read the appropriate section before selecting a response.

Materials & experimental systems

n/a	Involved in the study	
☐	☒	Antibodies
☐	☒	Eukaryotic cell lines
☒	☐	Palaeontology
☐	☒	Animals and other organisms
☐	☒	Human research participants
☒	☐	Clinical data

Methods

n/a	Involved in the study	
☒	☐	ChIP-seq
☐	☒	Flow cytometry
☒	☐	MRI-based neuroimaging

Antibodies

Antibodies used	All antibodies are described in Supplementary Table 5
Validation	All antibodies were obtained from commercial sources where they had previously been validated.

Eukaryotic cell lines

Policy information about cell lines

Cell line source(s)	Cell line information can be found in Methods subsection "Cell culture and reagents"
Authentication	Cell lines were purchased from and authenticated by ATCC.
Mycoplasma contamination	All cell lines tested negative for mycoplasma contamination. Please see Methods subsection: "Cell culture and reagents"
Commonly misidentified lines (See ICLAC register)	N/A

Animals and other organisms

Policy information about studies involving animals; ARRIVE guidelines recommended for reporting animal research

Laboratory animals	4-6 week old BALB/c mice were purchased from JAX labs.
Wild animals	N/A
Field-collected samples	N/A
Ethics oversight	Animal studies were approved under Protocol #2013-1020 by the Institutional Animal Care and Use Committee of Case Western Reserve University

Note that full information on the approval of the study protocol must also be provided in the manuscript.

Human research participants

Policy information about studies involving human research participants

Population characteristics	De-identified human breast cancer samples were assembled into a TMA. Age, sex, and race were all collected as part of this study.
Recruitment	N/A
Ethics oversight	Human breast cancer tissues were collected in accordance with the following IRB approved protocols: CASE 01-13-43C and CASE 7114.

Note that full information on the approval of the study protocol must also be provided in the manuscript.

Flow Cytometry

Plots

Confirm that:

☒ The axis labels state the marker and fluorochrome used (e.g. CD4-FITC).

☒ The axis scales are clearly visible. Include numbers along axes only for bottom left plot of group (a 'group' is an analysis of identical markers).

☒ All plots are contour plots with outliers or pseudocolor plots.

☒ A numerical value for number of cells or percentage (with statistics) is provided.

Methodology

Sample preparation	D2.A1 and D2.OR cells were obtained from Fred Miller as documented in the Methods subsection "Cell culture and reagents". Cells were harvested with Accutase and stained according to the protocol detailed in Methods subsection "CD49f/CD24 flow cytometry"
Instrument	Attune NxT Flow Cytometer
Software	Attune NxT Software Version 3.1
Cell population abundance	10,000 cells were analyzed for each sample type.
Gating strategy	Live (SSC-A and FSC-A) and single cells (FSC-A and FSC-H) were preliminarily gated. CD49f/CD24 gating is largely subjective and as such, upon selection of a gating strategy, gates remained static for all experiments. Gates were originally drawn on untreated D2.OR cell populations. See "Supplementary Fig. 8".

☒ Tick this box to confirm that a figure exemplifying the gating strategy is provided in the Supplementary Information.
